# Targeting KMT5C Suppresses Lung Cancer Progression and Enhances the Efficacy of Immunotherapy

**DOI:** 10.1002/advs.202407575

**Published:** 2025-03-24

**Authors:** Yunfeng Yuan, Qianyu Li, Guoquan Yan, Yifei Qian, Wenyun Guo, Songling Li, Fan Wang, Wanjing Shang, Zijun Zhu, Di Ge, Yanan Wang, Yanfeng Liu

**Affiliations:** ^1^ Department of Thoracic Surgery Zhongshan Hospital Fudan University Shanghai 200032 China; ^2^ Department of Liver Surgery Clinical Stem Cell Research Center Ren Ji Hospital Shanghai Jiao Tong University School of Medicine Shanghai 200127 China; ^3^ Institute of Biomedical Sciences Shanghai Medical College Fudan University Shanghai 200032 China; ^4^ Lymphocyte Biology Section Laboratory of Immune System Biology National Institute of Allergy and infectious Diseases National Institutes of Health Bethesda MD 20814 USA; ^5^ Department of Laboratory Medicine Ren Ji Hospital Shanghai Jiao Tong University School of Medicine Shanghai 200127 China; ^6^ Shanghai Engineering Research Center of Transplantation and Immunology Shanghai Institute of Transplantation Shanghai 200127 China

**Keywords:** immune checkpoint blockade therapy, immune evasion, lysine methyltransferase 5C, non‐small cell lung cancer, STING‐IRF3 signaling

## Abstract

The immune evasion is one major challenge for cancer immunotherapy. Despite considerable advancements in immune checkpoint blockade (ICB) therapies for the advanced non‐small cell lung cancer (NSCLC) patients, only a minority of patients receive long‐term survival benefit. Here, this work demonstrates that lysine methyltransferase 5C (KMT5C) is a crucial promoter of the NSCLC progression and immune evasion. This work first observes that upregulation of KMT5C in NSCLC correlated with cancer progression and poor patient prognosis. Notably, KMT5C knockdown in NSCLC cells suppress tumor growth and metastasis in mice. Mechanistically, this work demonstrates that KMT5C activated the DNA repair response to inhibit the STING‐IRF3 pathway, downstream type I IFN signaling, and CCL5 secretion, leading to the downregulation of CD8^+^ T cell infiltration and function in NSCLC, ultimately facilitating tumor immune evasion and tumor progression. Importantly, both the pharmacological inhibitor A196 and the genetic inhibition of KMT5C could synergize with anti‐PD‐1 therapy in the lung cancer mouse model. Clinically, high expression levels of KMT5C in patients with NSCLC are associated with a lower response rate and worse clinical outcomes to ICB therapy. Therefore, these findings identify a previously unknown functional link between KMT5C and tumor immune evasion, and demonstrate that targeting KMT5C may be a potential therapeutic approach for enhancing the efficacy of NSCLC patients to ICB therapy.

## Graphical abstract

This study reveals that KMT5C activates DNA damage repair to inhibit STING‐IRF3 pathway, downstream type I IFN signaling and CCL5 secretion, leading to the downregulation of CD8^+^ T cell infiltration and function in NSCLC, ultimately facilitating tumor progression. These findings demonstrate that targeting KMT5C may be a potential therapeutic approach for enhancing the efficacy of NSCLC patients to ICB therapy.

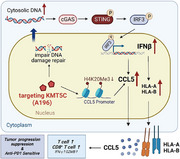

## 1 Introduction

Lung cancer is currently the leading cause of cancer‐associated deaths worldwide. Non‐small cell lung cancer (NSCLC) accounts for ≈80–85% of lung cancers and mainly encompasses lung adenocarcinoma (LUAD) and lung squamous cell carcinomas (LUSC), which have a very poor prognosis.^[^ ^1^ ^]^ Despite of rapid progress for novel treatment such as targeted therapy and immunotherapy in NSCLC, the 5‐year survival rate is still very low.^[^ ^2^, ^3^ ^]^ Currently, the immune checkpoint blockade (ICB) therapies, particularly the antibody therapies of the programmed death receptor 1 (PD‐1) and the programmed death ligand 1 (PD‐L1), are approved for the clinical therapy of advanced NSCLC patients.^[^ ^2^, ^4^, ^5^ ^]^ However, only a minority of patients receive long‐term survival benefits, owing to tumor heterogeneity and immune evasion. Therefore, investigating the mechanisms underlying tumor immune evasion is crucial for improving patient response rates to ICB therapy and the combination treatment strategies.

Epigenetic plasticity is crucial for tumor progression and immune evasion.^[^ ^6^ ^]^ Epigenetic regulators can affect cancer immunogenicity and immune responses by regulating genome stability.^[^ ^7^, ^8^ ^]^ Genomic instability is a hallmark of cancer development. DNA damage response (DDR) is the essential process for the perception and responses to DNA damage, which collectively encompasses DNA repair, cell cycle regulation, DNA replication, and apoptosis.^[^ ^9^ ^]^ In addition, DNA repair defects in cancer cells elevate the damaged cytosolic DNA, resulting in the activation of cyclin GMP‐AMP synthase/stimulator of IFN genes (cGAS‐STING) signaling and conferring a more sensitive phenotype to immunotherapy.^[^ ^10^, ^11^ ^]^ Thus, targeting epigenetic regulators may be a promising strategy to enhance the efficacy of immunotherapy in cancer.

Emerging evidence has shown that the methylation of histone H4 lysine 20 (H4K20) strongly regulates genomic integrity.^[^ ^9^, ^12^ ^]^ H4K20 is the only lysine multi‐methylation site on histone H4, that is highly conserved in eukaryotes.^[^ ^13^ ^]^ Lysine methyltransferase 5C (KMT5C) is reported to catalyze the third methylation of H4K20 and H4K20me3, which can induce transcriptional silencing.^[^ ^12^, ^14^ ^]^ Previously studies have reported that KMT5C plays a vital in cancer development, but its functions are complex, depending on the tissue context.^[^ ^9^, ^15^, ^16^, ^17^, ^18^, ^19^ ^]^ Currently, the role of KMT5C in NSCLC progression and immune evasion, including the mechanisms involved, remains unclear.

In this study, we have demonstrated that histone methyltransferase KMT5C may be a promising oncogenic protein for NSCLC progression. KMT5C can inhibit the STING‐IRF3 signaling‐mediated the type I IFN response by activating DNA damage repair, resulting in suppression of the NSCLC immune microenvironment and tumor progression. From a therapeutic viewpoint, our results have revealed that KMT5C inhibitor A196 not only shows a potential antitumor effect, but can also synergize with anti‐PD‐1 therapy in a lung cancer mouse model. Therefore, this study identifies a previously unknown functional link between KMT5C with tumor immune evasion and provides a potential therapeutic approach for the ICB therapy in patients with NSCLC.

## 2 Results

### 2.1 Upregulation of KMT5C in NSCLC Correlates with Cancer Progression and Poor Patient Prognosis

**Figure 1:**
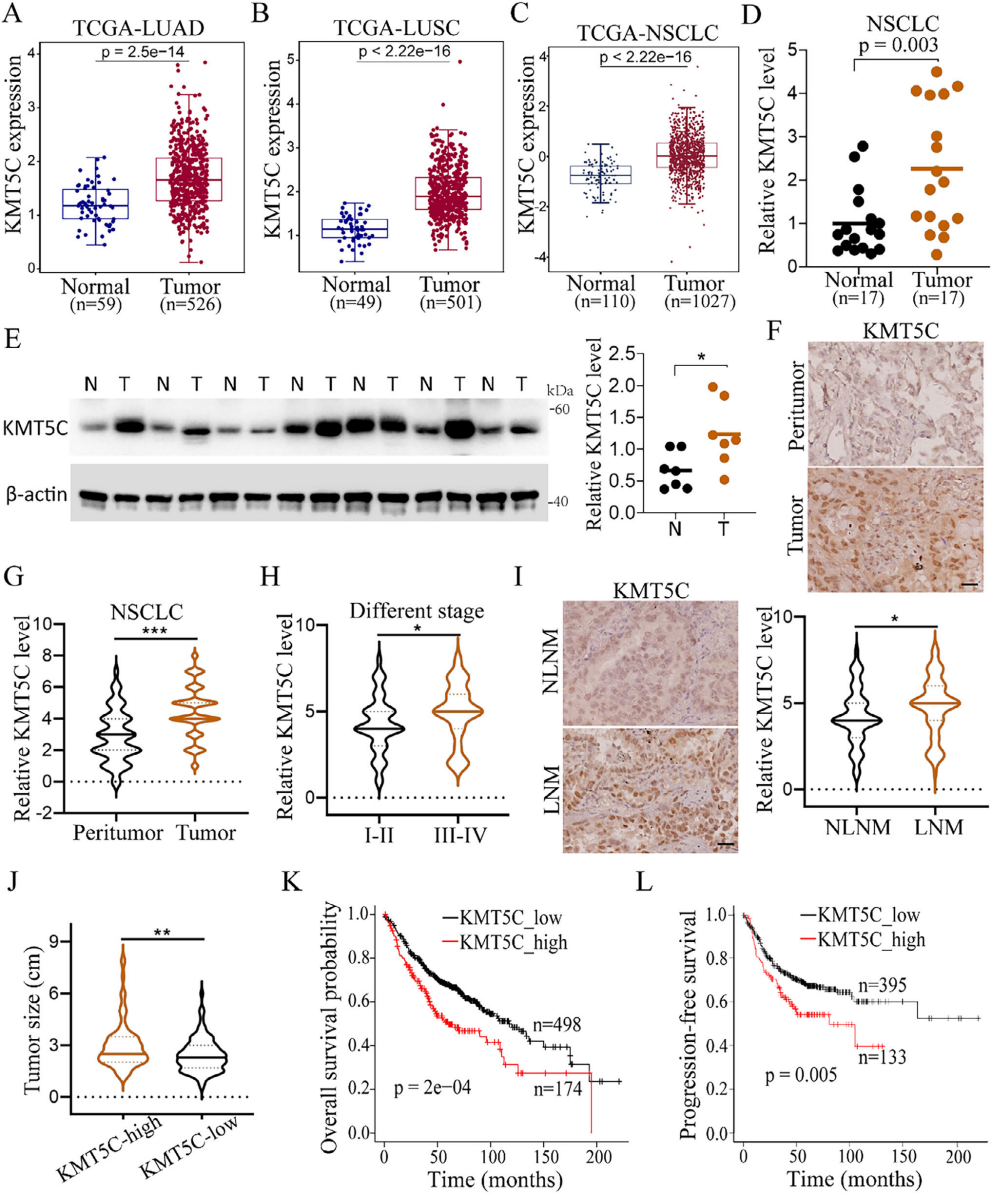
Upregulation of KMT5C in NSCLC is associated with cancer progression and poor prognosis. A–C) Comparison the mRNA level of KMT5C between normal and tumor tissues in the TCGA_LUAD (A), TCGA_LUSC (B) and TCGA_NSCLC (**C**) cohorts. D) Real‐time PCR analysis the expression level of KMT5C in NSCLC tumor tissues (n = 17) and paired non‐tumor tissues (n = 17). E) Western blot analysis and statistical the level of KMT5C in NSCLC tumor tissues (T, n = 7) and paired non‐tumor tissues (N, n = 7). F,G) Representative IHC staining images (F) and statistical data (G) of KMT5C level in NSCLC tumor tissues (n = 173) and peritumor tissues (n = 173). Scale bar, 50 µm. H) Relative IHC level of KMT5C between tumor different stage I‐II (n = 128) and III‐IV (n = 45) tissues. I) Representative IHC staining images and statistical data of KMT5C level in tumor tissues from NSCLC with lymph node metastasis (LNM, n = 67) and without lymph node metastasis (NLNM, n = 106). Scale bar, 50 µm. J) Comparison the tumor size between the NSCLC with KMT5C high expression level (KMT5C‐high) (n = 84) and KMT5C low expression group (KMT5C‐low) (n = 89). K, L) Kaplan‐Meier analysis of overall survival probability (K) and progression‐free survival probability (L) of KMT5C levels in lung cancer patients from Kaplan‐Meier plotter. The statistical significance was assessed using log‐rank test. For A–C and H–J, **s**tatistical significance was calculated using two‐tailed unpaired Student's *t*‐test. For D, E and G, **s**tatistical significance was calculated using two‐tailed paired Student's *t*‐test. *p < 0.05, **p < 0.01, ***p < 0.001.

To investigate the function of KMT5C in lung cancer progression, we first analyzed the expression levels of KMT5C in NSCLC, and found that KMT5C was significantly increased in tumor tissues compared with the normal tissues in the TCGA‐LUAD, TCGA‐LUSC and TCGA‐NSCLC databases (**Figure** **1**A–C). Similar results were also found in a lung cancer Gene Expression Omnibus (GEO) database (GSE30219) and a RNA‐seq data for LUSC^[^ ^20^ ^]^ (Figure S1A,B, Supporting Information). In addition, we further confirmed this finding in 17 paired NSCLC tumor tissues and non‐tumor tissues using the real‐time PCR assay, the result indeed showed that the mRNA level of KMT5C was significantly increased in the tumor tissues (Figure 1D). Similarly, the protein level of KMT5C was also upregulated in the tumor tissues relative to its adjacent counterparts (Figure 1E). Next, we further determined the expression of KMT5C in our in‐house NSCLC cohort using IHC staining, the expression level of KMT5C was observed to be upregulated in tumor tissues compared with that in peritumor tissues, and was also increased in advanced tumor stages (Figure 1F–H). Interestingly, the patients with lymph node metastasis (LNM) had higher KMT5C expression levels compared to those without lymph node metastasis (NLNM) (Figure 1I). Moreover, we also found that high KMT5C expression level was associated with a larger tumor size (Figure 1J). In addition, we further examined the relationship between KMT5C expression level and the clinical outcomes of patients with NSCLC, and Kaplan‐Meier plotter analyses revealed that patients with high KMT5C expression (KMT5C_high) showed shorter overall survival (OS) and progression‐free survival (PFS) than those with low expression of KMT5C (KMT5C_low) (Figure 1K,L). Similar results were consistently observed in several lung cancer cohorts (GSE29013, GSE50081 and GSE30219) (Figure S1C–F, Supporting Information). Overall, these findings suggest that KMT5C is a promising oncogenic protein in NSCLC.

### 2.2 KMT5C Promotes NSCLC Cell Proliferation, Migration and Invasion

**Figure 2:**
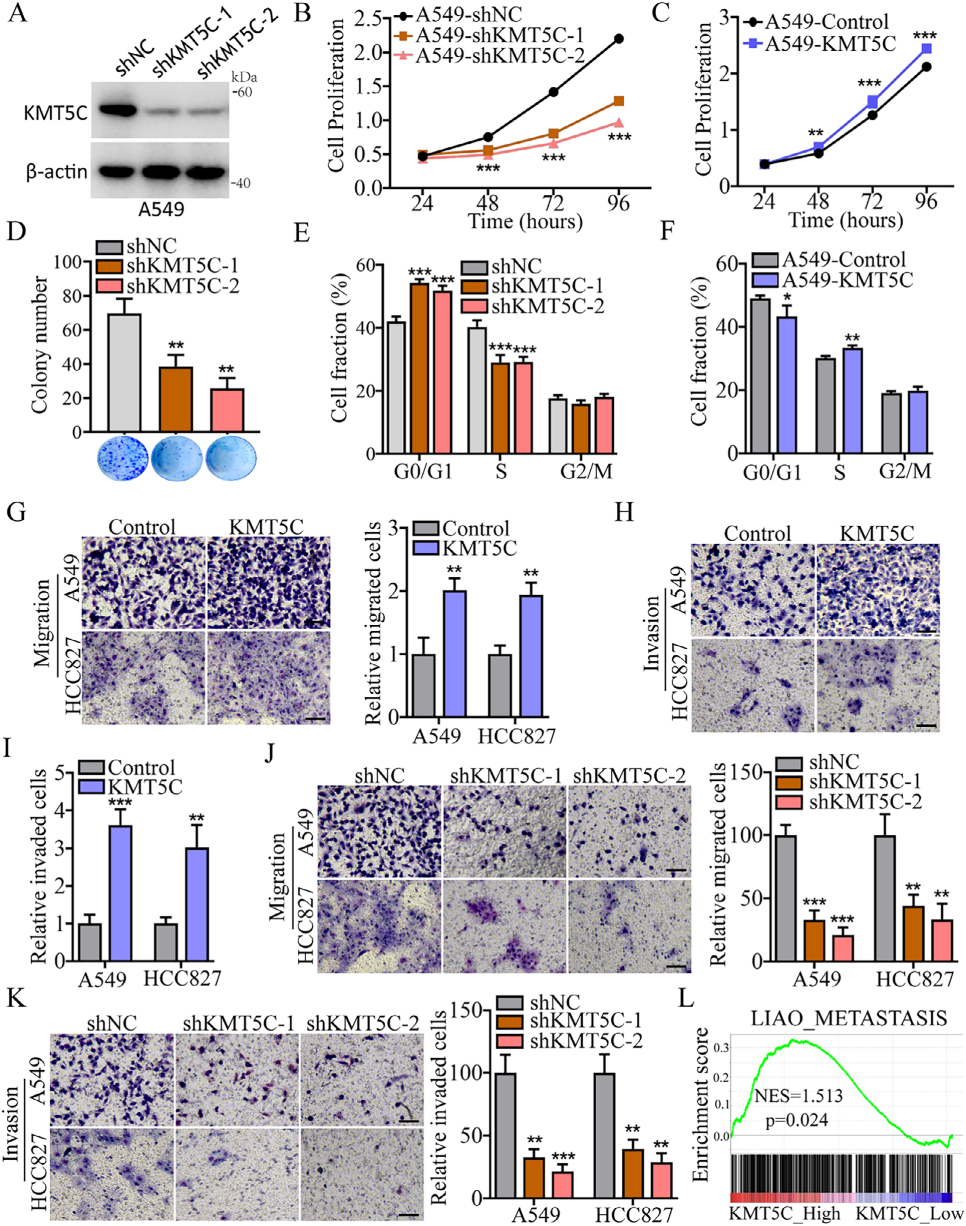
KMT5C promotes NSCLC cell proliferation, migration and invasion in vitro. A) Western blot analysis the A549 cells infected with the lentivirus expressing KMT5C knockdown shRNA (shKMT5C‐1 and shKMT5C‐1) or control shRNA (shNC). B) CCK8 assays analysis the cell proliferation from A549 cells infected with the indicated lentivirus (n = 6). C) CCK8 assays analysis the cell proliferation from A549 cells infected with control and KMT5C overexpression lentivirus (n = 6). D) Representative images and the statistical data of colony formation from the A549 cells infected with the indicated lentivirus (n = 3). E) Cell cycle analysis of A549 cells after stable knockdown of KMT5C (n = 4). F) Cell cycle analysis of A549 cells after KMT5C overexpression (n = 4). G) Representative the cell migration images and statistical data from A549 and HCC827 after KMT5C overexpression (n = 3). Scale bar, 50 µm. H,I) Representative the cell invasion images (H) and statistical data (I) from A549 and HCC827 after KMT5C overexpression (n = 3). Scale bar, 50 µm. J,K) Transwell assay analysis the cell migration (J) and invasion (K) from A549 and HCC827 after stable KMT5C knockdown (n = 3). Scale bar, 50 µm. L) GSEA shows the enrichment of LIAO_METASTASIS gene set in the TCGA_LUAD patients with KMT5C high expression group (KMT5C_High). Statistical significance was calculated using Permutation test. For D, E, J and K, statistical significance was calculated using one‐way ANOVA. For F, G and I, statistical significance was calculated using two‐tailed unpaired Student's t‐test. Data are presented as mean ± SD and *p < 0.05, **p < 0.01, ***p < 0.001.

To further explore the potential function of KMT5C in NSCLC progression, we first constructed stable KMT5C‐depleted A549 cells, and found that knockdown of KMT5C expression could reduce the tumor cell proliferative ability (**Figure** **2**A,B and Figure S2A, Supporting Information). Conversely, KMT5C overexpression increased A549 cell proliferation (Figure 2C and Figure S2B, Supporting Information). Consistently, the similar results were obtained in the tumor cell colony formation assay (Figure 2D and Figure S2C, Supporting Information). Moreover, we next examined whether KMT5C could affect the NSCLC cell cycle phase transition, and found that KMT5C knockdown could inhibit tumor cell cycle progression from the G0/G1 to S phase (Figure 2E and Figure S2D, Supporting Information). Also, KMT5C overexpression had the opposite effect (Figure 2F and Figure S2E, Supporting Information). Next, we further assessed the role of KMT5C in tumor metastasis using a transwell assay, and found that KMT5C overexpression indeed significantly enhanced the migration and invasion ability of A549 and HCC827 cells (Figure 2G–I and Figure S2F, Supporting Information). Conversely, knockdown the expression of KMT5C could remarkably reduce the tumor cell numbers of migration and invasion (Figure 2J,K and Figure S2G, Supporting Information). Similarly, GSEA revealed that the Liao_metastasis gene set was enriched in the KMT5C_High expression tumor tissues from TCGA_LUAD cohort (Figure 2L). Taken together, these findings indicate that KMT5C exerts pro‐tumor cell growth and metastasis in NSCLC.

### 2.3 Knockdown of KMT5C in NSCLC Suppresses Tumor Growth and Metastasis in Mice

**Figure 3:**
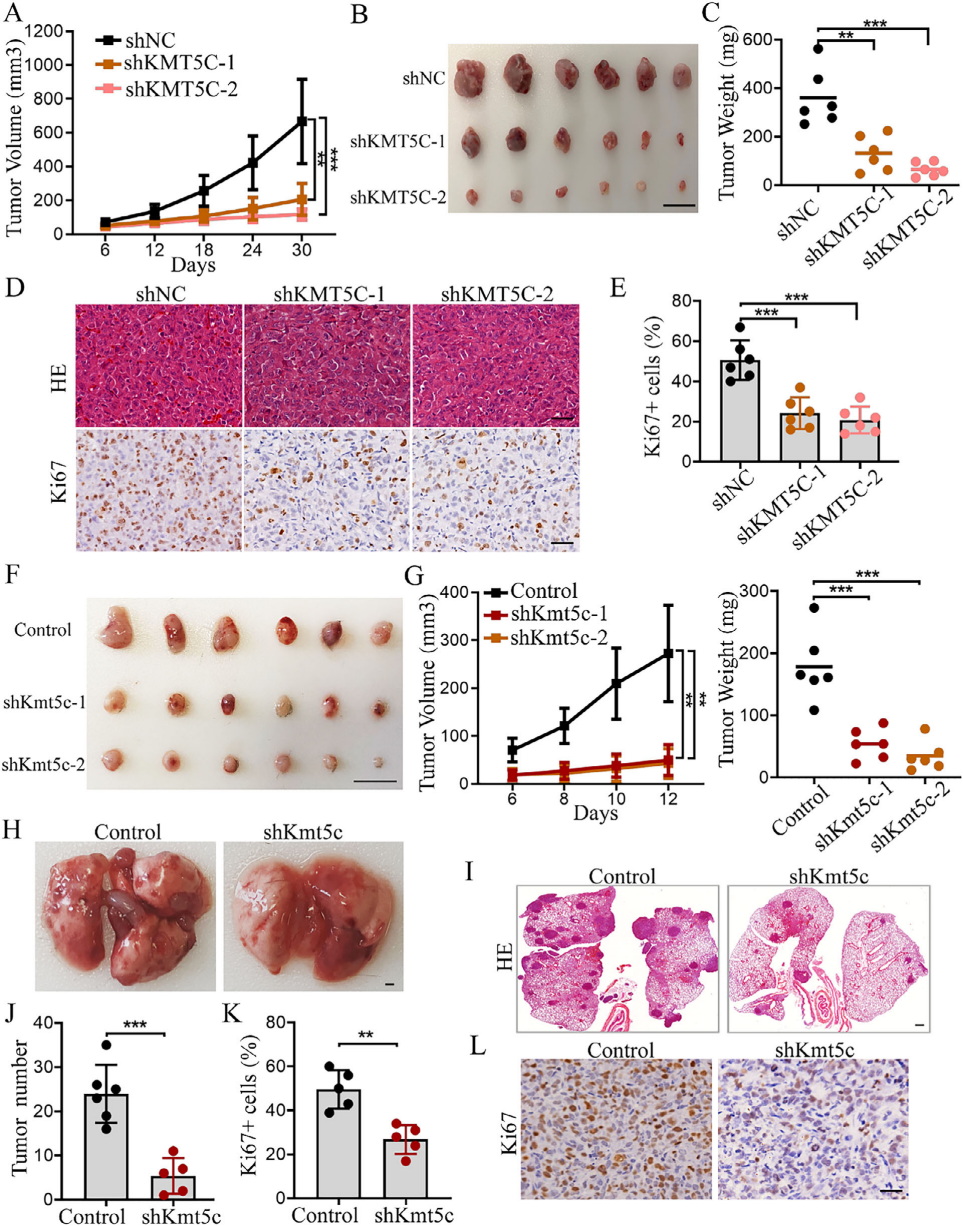
Knockdown of KMT5C in NSCLC suppresses tumor growth and metastasis in mice. A–C) A549 cells stably expressing the KMT5C knockdown shRNA (shKMT5C‐1 and shKMT5C‐2) and control shRNA (shNC) were subcutaneously injected in nude mice, respectively. Shown are average tumor volumes over time (A) and representative image (B) and weight (C) of tumors as the indicated (n = 6). Scale bar, 1 cm. D, E) Representative images of HE and IHC Ki‐67 staining (D), and the IHC scores of Ki‐67 expression (E) in the above tissues as indicated (n = 6). Scale bar, 50 µm. F,G) LLC cells stably expressing the Kmt5c knockdown shRNA (shKmt5c‐1 and shKmt5c‐2) and control shRNA (Control) were subcutaneously injected in C57BL/6 mice, respectively. Shown are the representative image of tumors (F), and statistical data of tumor volume and weight (**G**) as the indicated (n = 6). Scale bar, 1 cm. H–J) Representative images of lungs (H) from the C57BL/6 mice by tail‐vein injection of the LLC cells with control (Control) (n = 6) or Kmt5c knockdown (shKmt5c) (n = 5). The HE staining (I) and tumor number (J) of lungs were measured as indicated. Scale bar, 1 mm. K, L) The IHC scores of Ki‐67 expression (K) and representative image of IHC Ki‐67 staining (L) in the above tissues as indicated (n = 5). Scale bar, 50 µm. For A and G, statistical significance was calculated using two‐way ANOVA. For C, E and G, statistical significance was calculated using one‐way ANOVA. For J and K, statistical significance was calculated using two‐tailed unpaired Student's t‐test. Data are presented as mean ± SD and *p < 0.05, **p < 0.01, ***p < 0.001.

To further examine the role of KMT5C in vivo, we generated a tumor xenograft model using A549 cells with control or KMT5C knockdown. As expected, compared to the control group, KMT5C knockdown indeed could remarkably inhibit the tumor growth determined by the lower tumor volume and tumor weight (**Figure** **3**A–C). Moreover, IHC analysis revealed that the Ki67 positive cells were reduced in KMT5C knockdown tumors (Figure 3D,E), and the knockdown effect of KMT5C in vivo was confirmed by detecting the levels of KMT5C and its substrate H4K20me3 (Figure S3A,B, Supporting Information). Next, we further confirmed the function of KMT5C in vivo using the mouse lung cancer cell line LLC. Consistently, the Kmt5c knockdown group had the lower tumor volume and weight than the control group (Figure 3F,G). In addition, the Ki67 positive cell numbers were decreased in Kmt5c knockdown tumors (Figure S3C,D, Supporting Information). To further examine the role of KMT5C in tumor metastasis in vivo, we generated a mouse lung metastasis model by tail vein injection of the LLC cells with control or Kmt5c knockdown. The results revealed that Kmt5c knockdown significantly reduced the tumor number in the mouse lung (Figure 3H–J). Additionally, the Kmt5c knockdown group had lower Ki67 positive cell numbers than the control group (Figure 3K,L). These results demonstrate the crucial role of KMT5C in facilitating tumor growth and metastasis in vivo.

### 2.4 KMT5C Regulates the DNA Damage Response to Suppress the STING‐IRF3 Pathway, Downstream Type I IFN Signaling and Reduces CCL5 Expression

**Figure 4:**
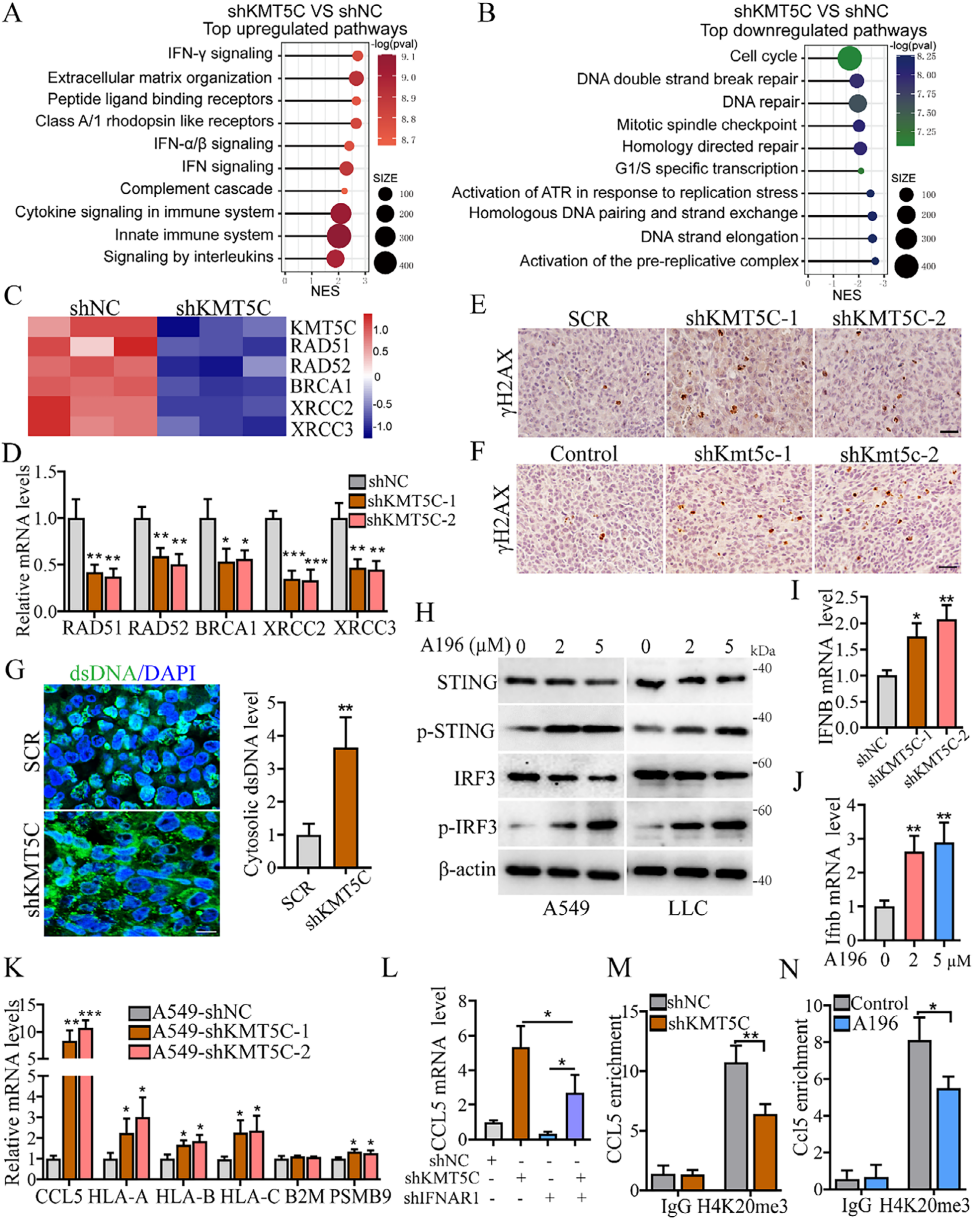
KMT5C knockdown impairs DNA damage response to activate the STING‐IRF3, downstream type I IFN signaling and induces CCL5 expression. A,B) GSEA analysis the RNA‐seq data from A549 cells with KMT5C knockdown (shKMT5C) or control (shNC) (n = 3). Shown are the images of top upregulated pathways (A) (shKMT5C VS shNC) and top downregulated pathways (B) (shKMT5C VS shNC). **C** Heatmap demonstration of the gene expression levels of RAD51, RAD52, BRCA1, XRCC2 and XRCC3 in the RNA‐seq data from A549 cells after stably KMT5C knockdown as indicated. D) Real‐time PCR analysis the relative mRNA levels in A549 cells as indicated (n = 3). E) Representative images of IHC γH2AX staining in tumor tissues from A549‐shNC or A549‐shKMT5C‐1&‐2 mice group. Scale bar, 50 µm. F) Representative images of IHC γH2AX staining in tumor tissues from LLC‐shKmt5c or Control mice group. Scale bar, 50 µm. G) Confocal images and quantification of cytosolic dsDNA in tumor tissues from A549‐shKMT5C or Control group (n = 3). H) Western blot analysis the levels of STING, p‐STING, IRF3 and p‐IRF3 in the A549 and LLC cells upon A196 treatment as indicated. I) Real‐time PCR analysis the levels of IFNB in A549 cells as indicated (n = 3). J) Real‐time PCR analysis the levels of Ifnb in LLC cells upon A196 treatment as indicated (n = 3). K) Real‐time PCR analysis the relative mRNA levels of CCL5, HLA‐A, HLA‐B, HLA‐C, B2M and PSMB9 from A549 cells after stably KMT5C knockdown as indicated (n = 3). L) Real‐time PCR analysis the relative mRNA levels of CCL5 in A549 cells infected with the indicated lentivirus (n = 3). M) ChIP‐qrtPCR analysis the sonicated chromatins immunoprecipitated from A549 cells (shNC and shKMT5C) by anti‐H4K20me3 antibody or IgG control (n = 3). N) ChIP‐qrtPCR analysis the sonicated chromatins immunoprecipitated from LLC cells (with or without A196 treatment) by anti‐H4K20me3 antibody or IgG control (n = 3). For D, I, J, K and L, statistical significance was calculated using one‐way ANOVA. For G, M and N, statistical significance was calculated using two‐tailed unpaired Student's *t‐*test. Data are presented as mean ± SD and *p < 0.05, **p < 0.01, ***p < 0.001.

To explore the potential molecular mechanism of KMT5C promoting NSCLC progression, we first performed RNA‐seq using A549 cells with control or KMT5C knockdown. Gene set enrichment analysis (GSEA) revealed that interferon‐related signaling pathways and cytokine signaling were significantly enriched in the KMT5C knockdown group (**Figure** **4**A), but several DNA damage response (DDR) pathways were remarkably compromised such as the cell cycle, homologous recombination, DNA repair and DNA replication (Figure 4B). Consistently, the inflammatory response, interferon alpha response, interferon gamma response and chemokine signaling pathway were significantly enriched in the KMT5C low expression group from the TCGA_LUAD cohort, while the gene sets of homologous recombination, DNA replication, cell cycle‐related G2M checkpoint and proliferation‐related E2F targets were enriched in the LUAD patients with high expression of KMT5C (Figure S4A,B, Supporting Information). These findings suggest that KMT5C may participate in regulation of DDR and interferon response signaling pathways in NSCLC. Next, we further examined the RNA‐seq data, which showed that the expression of several key genes (RAD51, RAD52, BRCA1, XRCC2 and XRCC3) involved in DNA double strand breaks repair was reduced upon KMT5C knockdown (Figure 4C). In addition, a real‐time PCR assay revealed a similar result in the KMT5C knockdown A549 cells (Figure 4D). Moreover, we examined whether KMT5C could affect the expression of the E2F1, the known transcription factor in regulating the expression of DDR‐related genes.^[^ ^21^, ^22^, ^23^ ^]^ The western blot and real‐time PCR experiments showed that KMT5C knockdown could decrease E2F1 protein stability in A549 cells (Figure S4C,D, Supporting Information). Given that the ataxia telangiectasia mutated (ATM) kinase plays a crucial role of DNA repair in response to DNA double‐strand breaks (DSBs) and can enhance E2F1 protein stabilization,^[^ ^21^, ^24^ ^]^ we further determined whether KMT5C could regulate ATM activity. The results showed that KMT5C knockdown in A549 cells inhibited the activity of ATM kinase, as determined by the decreased phosphor‐ATM (p‐ATM), and could further inhibited its activity on DSBs induction by hydroxyurea (HU) (Figure S4E, Supporting Information). These results suggest that the ATM‐mediated E2F1 protein stabilization may contribute to the role of KMT5C in regulating the expression of DDR‐related genes. Furthermore, we investigated whether KMT5C could regulate DNA damage repair in NSCLC. As expected, we examined the level of γH2AX, a common marker of DSBs, by IHC assay, and found that the γH2AX positive cells were significantly upregulated in the KMT5C knockdown human lung tumor tissues (Figure 4E and Figure S4F, Supporting Information). Furthermore, a similar result was observed in Kmt5c knockdown mouse lung tumor tissues (Figure 4F and Figure S4G, Supporting Information). We also examined cytosolic double‐stranded DNA (dsDNA) levels upon KMT5C inhibition and found that the cytosolic dsDNA levels were significantly increased in the KMT5C knockdown human lung tumor tissues and Kmt5c knockdown mouse lung tumor tissues (Figure 4G and Figure S4H, Supporting Information). These findings indicate that KMT5C can indeed promote DNA damage repair in lung cancer cells.

Given that inhibition of DDR can induce the elevation of the cytosolic dsDNA, resulting in the activation of cGAS‐STING pathway and downstream type I IFN signaling,^[^ ^25^, ^26^, ^27^ ^]^ we further determined whether DNA repair driven by KMT5C could inhibit the cGAS‐STING signaling activity. As expected, KMT5C knockdown in A549 cells resulted in the activation of GAS‐STING signaling (Figure S4I, Supporting Information), as determined by the elevated phospho‐STING (p‐STING) and phospho‐interferon regulatory factor 3 (p‐IRF3), the common activity indicator of GAS‐STING signaling.^[^ ^26^, ^27^ ^]^ Consistently, we used A196,^[^ ^12^ ^]^ a substrate‐competitive inhibitor of KMT5C, to treat the A549 and LLC cells, and found the similar results in the activation of GAS‐STING signaling upon A196 treatment (Figure 4H). Similarly, GSEA found that the ISHIKAWA_STING_SIGNALING was enriched in the KMT5C knockdown group (Figure S4J, Supporting Information). We further examined the role of KMT5C inhibition in activating type I IFN signaling, and the results revealed that KMT5C knockdown indeed could significantly upregulated IFNB expression in A549 cells (Figure 4I). Also, the similar result was observed in the LLC cells upon A196 treatment (Figure 4J). In addition, the antigen processing and presentation gene set was also enriched in KMT5C knockdown group (Figure S4K, Supporting Information). These results indicate that KMT5C can reduce the activity of STING‐IRF3 signaling to inhibit the innate immune system. Furthermore, the volcano plot revealed that the CCL5, a common downstream chemokine of STING‐IRF3 signaling,^[^ ^26^, ^28^ ^]^ was highly enriched in the differentially expressed genes upon KMT5C knockdown (Figure S4L, Supporting Information). Also, real‐time PCR assays showed that the expression of CCL5 and several key genes (HLA‐A, HLA‐B, HLA‐C, B2M and PSMB9) involved in antigen presentation and processing was upregulated in KMT5C knockdown A549 cells (Figure 4K). Conversely, overexpression of KMT5C in HCC827 cells reduced the mRNA levels of the above genes (Figure S4M, Supporting Information). Furthermore, we used A196 to treat the LLC cells, and found that the expression of Ccl5 was significantly increased upon A196 treatment (Figure S4N, Supporting Information). Given that the type I IFN can induce CCL5 expression,^[^ ^25^, ^26^, ^27^ ^]^ we investigated whether the upregulation of CCL5 expression by KMT5C inhibition was dependent on the type I IFN signaling. To this end, we examined the expression of CCL5 in the inhibition of type I IFN signaling through IFNAR1 knockdown A549 cells and found that IFNAR1 knockdown only partially impaired the regulation of CCL5 caused by A196 treatment or KMT5C knockdown (Figure 4L and Figure S4O,P, Supporting Information). Next, we investigated whether the histone methyltransferase activity of KMT5C also contributes to the regulation of CCL5. Thus, we detected the H4K20me3 levels in the CCL5 promoter using chromatin immunoprecipitation (ChIP) assay with anti‐H4K20me3 and anti‐IgG antibodies. As expected, the level of H4K20me3 on the CCL5 promoter was remarkably decreased in KMT5C knockdown A549 cells (Figure 4M). Also, the similar result was observed in the LLC cells upon A196 treatment (Figure 4N). These results suggest that the STING‐IRF3 pathway‐mediated type I IFN signaling activation and KMT5C inhibition‐mediated demethylation of H4K20me3 both contribute to the CCL5 regulation. Taken together, these data demonstrate that inhibition of KMT5C can induce DDR defects in NSCLC, that leading to activation of the STING‐IRF3 pathway, downstream type I IFN signaling and induce CCL5 expression.

### 2.5 KMT5C Knockdown Mediated the Improvement of Tumor Immune Microenvironment May Mainly Depend on CCL5

**Figure 5:**
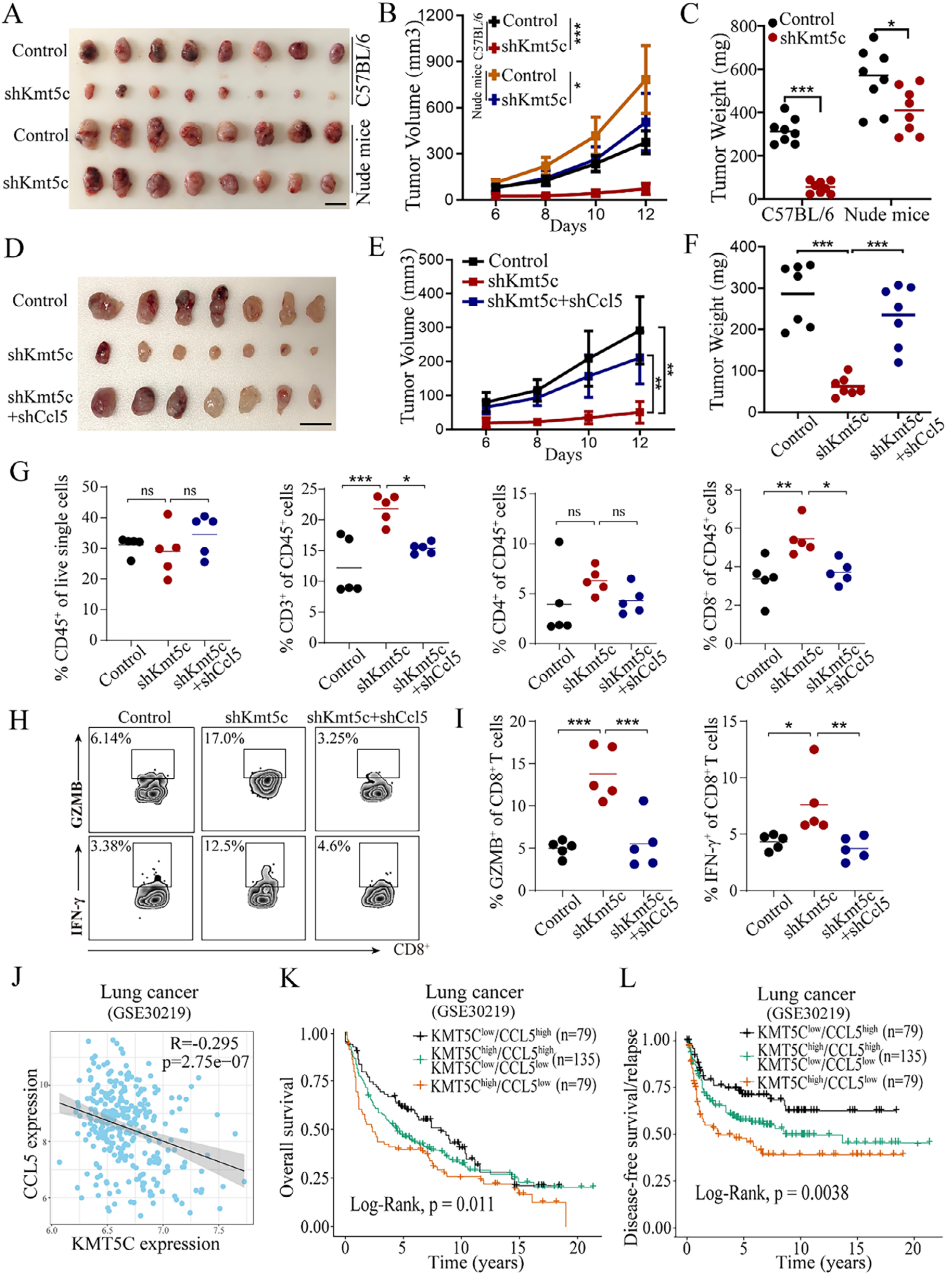
CCL5 is required for KMT5C knockdown‐mediated the improvement of tumor immune microenvironment. A–C) LLC cells with Control or Kmt5c knockdown were subcutaneously injected in C57BL/6 mice and nude mice. Shown are the representative image of tumors (A), and statistical data of tumor volume (B) and weight (C) as the indicated (n = 8). Scale bar, 1 cm. D–F) LLC cells stably expressing the indicated shRNA were subcutaneously injected in C57BL/6 mice, respectively. Shown are the representative image of tumors (D), and statistical data of tumor volume (E) and weight (F) as the indicated (n = 7). Scale bar, 1 cm. G) Relative proportions of CD45^+^, CD3^+^, CD4^+^ and CD8^+^ T cells in tumor tissues were analyzed by flow cytometry (n = 5). H) Gating strategies for analysis of cytotoxic function of CD8^+^ T cells by flow cytometry. I) Relative proportions of IFN‐γ^+^ and GAMB^+^ of CD8^+^ T cells in tumor tissues were analyzed by flow cytometry (n = 5). J) Correlation analysis of the expression of KMT5C and CCL5 in lung cancer tissues from a GEO database (GSE30219). The Pearson coefficient was used to evaluate correlations. K,L) Kaplan‐Meier analysis of overall survival (K) and disease‐free survival/relapse (L) in lung cancer patients (GSE30219) according to combined expression status of KMT5C and CCL5 as indicated. The statistical significance was assessed using log‐rank test. For B and E, statistical significance was calculated using two‐way ANOVA. For C, F, J and I, statistical significance was calculated using one‐way ANOVA. Data are presented as mean ± SD and ns. not significant, *p < 0.05, **p < 0.01, ***p < 0.001.

Given that tumor cell‐expressed CCL5 is essential for T cell infiltration,^[^ ^29^, ^30^ ^]^ we wondered to examine the contribution of T cells to the tumor‐suppressive effect of KMT5C knockdown in NSCLC. To this end, we compared the tumor growth of LLC cells with control or Kmt5c knockdown in T cell‐deficient nude mice and wild‐type C57BL/6 mice. We found that the antitumor effect of shKmt5c was stronger in C57BL/6 mice than in nude mice (**Figure** **5**A–C), indicating that the T cells participated in controlling tumor growth in the shKmt5c group. Next, we examined the contribution of CCL5‐mediated T cell infiltration to the tumor‐suppressive effect of KMT5C knockdown. To this end, we first constructed the double Kmt5c and Ccl5 knockdown LLC cells to investigate whether the speed of tumor growth could be rescued compared to that in Kmt5c single knockdown LLC cells. The expression levels of Kmt5c and Ccl5 were determined by real‐time PCR assay (Figure S5A,B, Supporting Information). CCK assays showed that Ccl5 knockdown could not influence the cell proliferation phenotype of shKmt5c in vitro (Figure S5C, Supporting Information), suggesting that CCL5 may mainly contribute to the role of KMT5C in regulating the tumor immune microenvironment. Moreover, we further confirmed this result in nude mice, and found that that Ccl5 knockdown could not significantly influence the tumor growth phenotype of shKmt5c mice group in nude mice (Figure S5D–F, Supporting Information). In addition, we generated a tumor xenograft model in C57BLC/6 mouse, and found that the phenotypes of the shKmt5c mice group were almost rescued by Ccl5 knockdown (Figure 5D–F). Similarly, the reduction in the Ki67 positive cell number mediated by the Kmt5c knockdown was also rescued by Ccl5 knockdown (Figure S5G,H, Supporting Information). Next, we further examined the tumor immune microenvironment of the above tumor xenograft mouse model by flow cytometry assay and the results showed that the proportions of CD3^+^ T cells and CD8^+^ T cells were increased in the shKmt5c group, and the production of cytotoxic molecules IFN‐γ and GZMB were also upregulated in the tumor‐infiltrating CD8^+^ T cells of the shKmt5c group, but these improvements in the tumor immune microenvironment of the shKmt5c group could be almost abolished by Ccl5 knockdown (Figure 5G–I and Figure S5I, Supporting Information). Furthermore, IHC analysis also revealed that Kmt5c knockdown significantly enhanced the infiltration of CD3^+^ T and CD8^+^ T cells, and upregulated the expression level of IFN‐γ, but these improvements in the immune microenvironment of the shKmt5c group could be almost abolished by Ccl5 knockdown (Figure S5J,K, Supporting Information), suggesting that CCL5 is required for the role of KMT5C in regulating tumor immune microenvironment.

In addition, we also examined the relationship between KMT5C and CCL5 expression in clinical lung cancer samples. Pearson correlation analysis revealed that KMT5C expression had a negatively associated with CCL5 levels in the lung cancer specimens (GSE30219) (Figure 5J). Moreover, Kaplan‐Meier analysis revealed that lung cancer patients with KMT5C^high^/CCL5^low^ showed the shortest overall survival and disease‐free survival/relapse, whereas the KMT5C^low^/CCL5^high^ group had the best clinical outcomes (Figure 5K,L). Overall, these results indicate that the KMT5C‐CCL5 axis plays an important role in NSCLC progression.

### 2.6 KMT5C Inhibitor can Enhance the Efficacy of Anti‐PD1 Therapy in NSCLC

**Figure 6:**
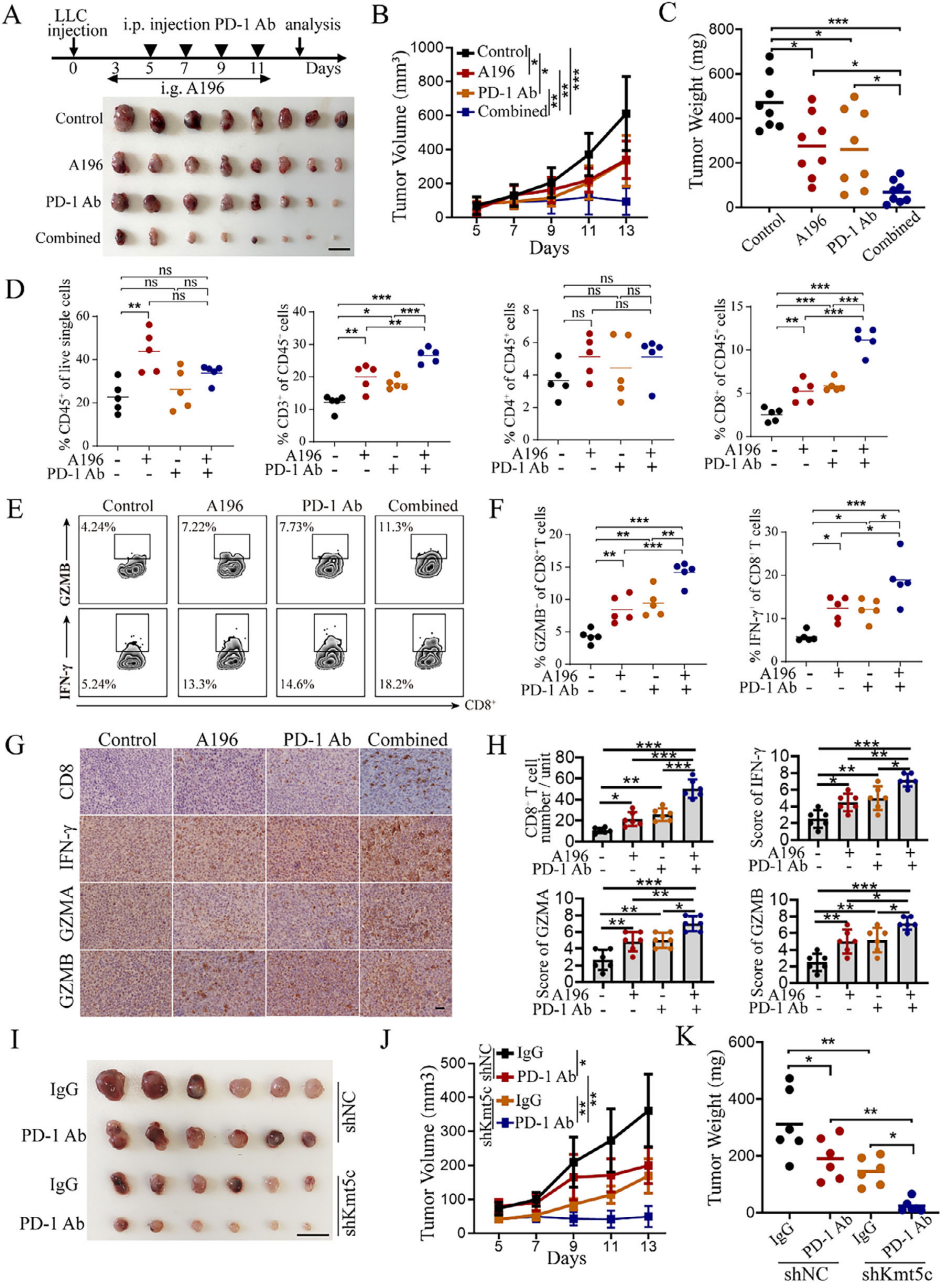
KMT5C inhibitor potentiates the response of NSCLC to anti‐PD‐1 therapy. A–C) LLC cells were subcutaneously injected in C57BL/6 mice (n = 8 mice/group). After 3–5 days post‐injection, mice were treated with vehicle control and A196 (20 mg per kg daily, intragastrically (i.g)), anti‐PD‐1 antibody (100 µg per time, every 2 days for a total of 4 intraperitoneal injection (i.p)) or a combined respectively until the end of experiments. Shown are the representative image of tumors (A), and statistical data of tumor volume (B) and weight (C) as the indicated (n = 8). Scale bar, 1 cm. D) Relative proportions of CD45^+^, CD3^+^, CD4^+^ and CD8^+^ T cells in tumor tissues were analyzed by flow cytometry (n = 5). E) Gating strategies for analysis of cytotoxic function of CD8^+^ T cells by flow cytometry. F) Relative proportions of IFN‐γ^+^ and GAMB^+^ of CD8^+^ T cells in tumor tissues were analyzed by flow cytometry (n = 5). G,H) Representative images of IHC CD8, IFN‐γ, GZMA and GZMB staining (G) and the statistical data (H) of numbers of the positive cells of CD8, and the IHC scores of IFN‐γ, GZMA and GZMB expression in the tumor tissues of mouse as indicated (n = 6). Scale bar, 50 µm. I–K) LLC cells stably expressing the Kmt5c knockdown shRNA (shKmt5c) and control shRNA (shNC) were subcutaneously injected in C57BL/6 mice respectively with or without anti‐PD‐1 antibody treatment. Shown are the representative image of tumors (I), and statistical data of tumor volume (J) and weight (K) as the indicated (n = 6). Scale bar, 1 cm. For B and J, statistical significance was calculated using two‐way ANOVA. For C, D, F, H and K, statistical significance was calculated using one‐way ANOVA. Data are presented as mean ± SD and ns. not significant, *p < 0.05, **p < 0.01, ***p < 0.001.

Given that KMT5C knockdown can improve the immunosuppressive tumor microenvironment, we wondered whether KMT5C inhibition could potentiate the response of NSCLC to immunotherapy. To this end, we first constructed a tumor xenograft model using LLC cells. Mice were respectively administered the vehicle (control), KMT5C inhibitor (A196), PD‐1 antibody (PD‐1 Ab), or a combination of A196 and PD‐1 antibody (combined), and the results revealed that KMT5C inhibitor A196 could reduce the tumor volume and weight compared with the control group, the combination therapy group displayed more effective in inhibiting tumor progression than A196 or PD‐1 Ab alone, as indicated by the lower tumor volume, tumor weight, and fewer Ki67 positive cells following the combined treatment (**Figure** **6**A–C and Figure S6A, Supporting Information). This suggests that pharmacological inhibition of KMT5C can synergize with anti‐PD‐1 therapy in the lung cancer mouse model. Furthermore, flow cytometry analysis revealed that A196 combined with anti‐PD‐1 treatment could significantly increase the cell infiltration of CD3^+^ T cells and CD8^+^ T cells, and upregulate the levels of CD8^+^ T cell cytotoxic markers IFN‐γ and GZMB (Figure 6D–F). Moreover, IHC analysis revealed the similar results in tumor tissues from tumor xenograft mice (Figure 6G,H). In addition, we further examined whether the genetic inhibition of KMT5C could also promote the efficacy of anti‐PD‐1 therapy in NSCLC. We established the lung cancer mouse model using the LLC cells with control or Kmt5c knockdown and divided the mice into two groups. The mice were then respectively administered IgG and PD‐1 antibodies 5 days after the LLC cells injection. Consistently, the results showed that Kmt5c knockdown also sensitized lung cancer cells to anti‐PD‐1 therapy, as the tumor volume and weight were further reduced upon the combination of Kmt5c knockdown and PD‐1 antibody treatment compared to the kmt5c knockdown or anti‐PD‐1 therapy alone (Figure 6I–K). Furthermore, flow cytometry analysis found higher levels of CD3^+^ T cells and CD8^+^ T cells infiltration, and the CD8^+^ T cell cytotoxic markers (IFN‐γ and GZMB) in the combination of Kmt5c knockdown and PD‐1 antibody treatment group (Figure S6B, Supporting Information). IHC analysis revealed the similar results in the combination of Kmt5c knockdown and PD‐1 antibody treatment group (Figure S6C, Supporting Information). In addition, GSEA also revealed that the gene sets up in responders treated with ICB therapies were significantly enriched in the KMT5C knockdown group (Figure S6D, Supporting Information). Together, these data suggest that either the pharmacological inhibitor A196 or the genetic inhibition of KMT5C can not only restore CD8^+^ T cell function and suppress tumor growth, but can also enhance the efficacy of anti‐PD1 therapy in NSCLC.

### 2.7 KMT5C High Expression in NSCLC Correlates with Therapy Resistance and Worse Prognosis to ICB Therapy

**Figure 7:**
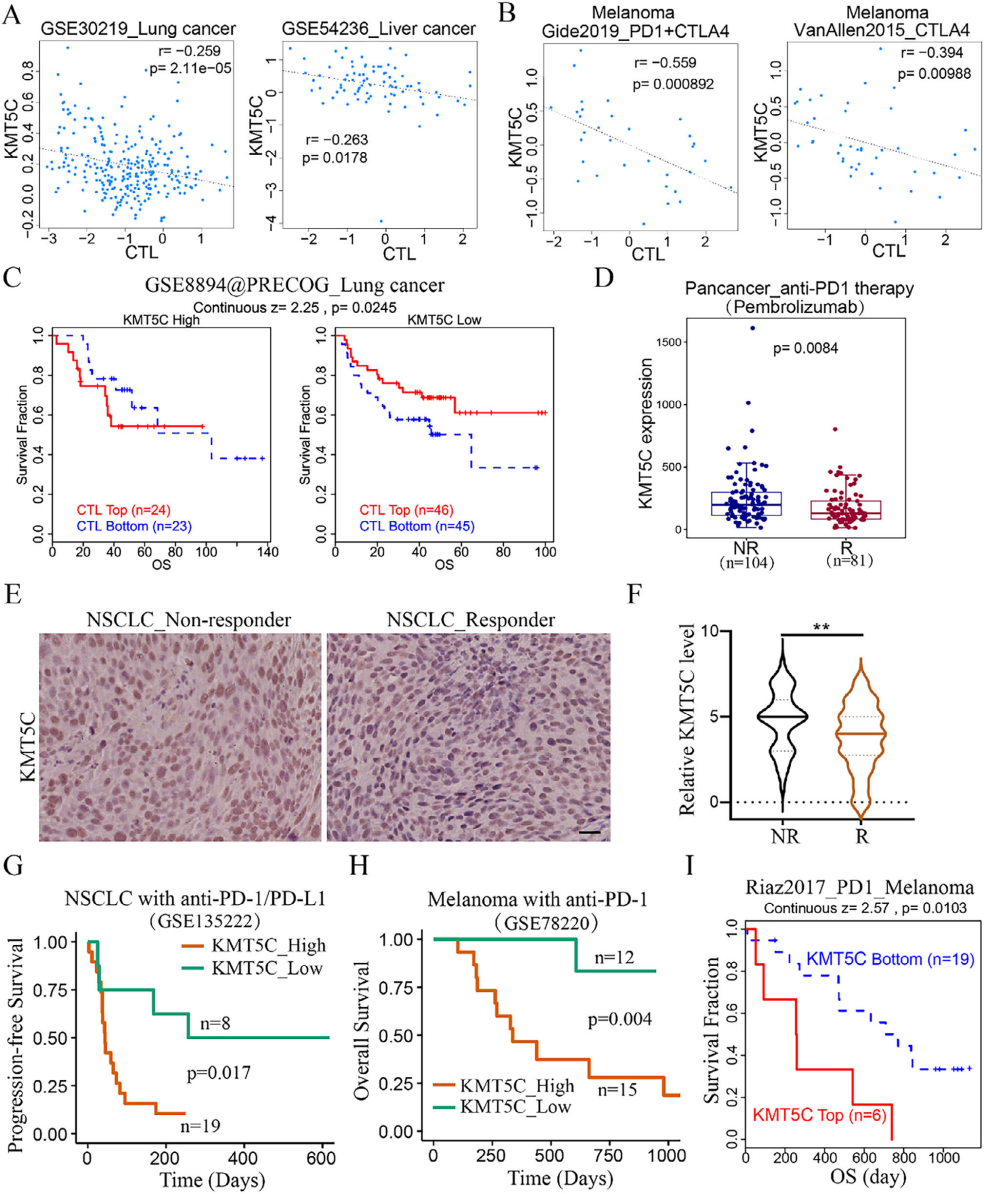
KMT5C high expression in NSCLC is associated with immunotherapy resistance and worse prognosis to ICB therapy. A,B) Correlation analysis of the cytotoxic T lymphocyte (CTL) level and the expression of KMT5C in lung cancer (GSE30219) and liver cancer (GSE54236) (A), and two melanoma ICB therapy cohorts (Gide2019_PD1+CTLA4 and VanAllen2015_CTLA4) (B) from the TIDE database (http://tide.dfci.harvard.edu/). The Pearson coefficient was used to evaluate correlations. C) Kaplan‐Meier analysis of the association between CTL levels with overall survival (OS) in lung cancer patients with KMT5C high or low expression group from the TIDE database (http://tide.dfci.harvard.edu/). D) The expression level of KMT5C in pancancer_anti‐PD1 therapy non‐responder (NR) and responder (R) from the ROC plotter database (http://www.rocplot.com/). E,F) Representative images of IHC KMT5C staining (E) and the IHC scores of KMT5C expression (F) in the NSCLC tumor tissues from ICB therapy non_responder (n = 39) or responder (n = 50). Scale bar, 50 µm. G) Kaplan‐Meier analysis of progression‐free survival and KMT5C expression levels in NSCLC with anti‐PD‐1/PD‐L1 treatment cohort (GSE135222) as indicated. The statistical significance was assessed using log‐rank test. H) Kaplan‐Meier analysis of overall survival and KMT5C levels in melanoma with anti‐PD‐1 therapy cohort (GSE78220). I) Survival analysis of overall survival (OS) of KMT5C levels in melanoma patients receiving anti‐PD‐1 therapy (Riaz2017_PD1_Melanoma) from TIDE database (http://tide.dfci.harvard.edu/). For D and F, statistical significance was calculated using two‐tailed unpaired Student's *t*‐test and **p < 0.01.

Next, we wondered whether KMT5C expression level could predict immune checkpoint blockade (ICB) therapy response in cancer patient samples. Given the level of cytotoxic T lymphocyte (CTL) affects ICB efficacy, we first examined the relationship between KMT5C expression and CTL level in cancer tissues. As expected, KMT5C expression was negatively associated with CTL levels in several cancer tissues, including lung cancer (**Figure** **7**A and Figure S7A, Supporting Information). Similarly, these results were observed in the melanoma upon ICB treatment cohorts (Figure 7B). Next, we further examined whether KMT5C expression level could affect CTL‐mediated patient clinical outcomes. The result revealed that the CTL high‐expression group displayed a longer survival time in lung cancer patients with KMT5C low expression (Figure 7C). Similar results were also found in several cancer types in the TIDE database (Figure S7B–D, Supporting Information). Furthermore, we analysed whether the expression level of KMT5C affected the response to ICB therapy. We found that KMT5C expression was upregulated in non‐responder (NR) from the pancancer_anti‐PD1 therapy cohort compared to the responder (R) group (Figure 7D). Similarly, IHC analysis revealed that the expression of KMT5C was also higher in the tissues of non‐responder to NSCLC_ICB therapy (Figure 7E,F). Next, we sought to determine whether KMT5C could affect ICB therapy‐mediated patient clinical outcomes. Consistently, NSCLC patients with KMT5C high expression indeed showed shorter progression‐free survival upon ICB treatment (Figure 7G). Similarly, patients with KMT5C high expression had worse clinical outcomes in the melanoma and pancancer datasets (Figure 7H,I and Figure S7E–G, Supporting Information). Taken together, these findings suggest that high KMT5C levels can be a biomarker for poor response to ICB therapy.

## 3 Discussion

Although cancer immunotherapy has achieved considerable advancements in recent years, the immune evasion is a major challenge in improving the clinical effectiveness of immunotherapy.^[^ ^31^, ^32^, ^33^ ^]^ Here, we show that KMT5C has a crucial role in regulation of tumor immune evasion in lung cancer. Although previously studies have reported that KMT5C plays an important role in cancer development, the relationship between KMT5C and immunity regulation remains unclear. The current work is the first to reveal the regulatory effect of KMT5C in the tumor immunity microenvironment. Consistent with its role, we demonstrate that KMT5C activates the DNA repair response to inhibit the STING‐IRF3 pathway, downstream type I IFN signaling and CCL5 secretion, leading to the downregulation of CD8^+^ T cell infiltration and function in NSCLC, ultimately facilitating tumor immune evasion and tumor progression. Our study further indicates that the ATM‐mediated E2F1 protein stabilization may contribute to the role of KMT5C in regulating DDR. In addition to ATM promoting E2F1 stability, P/CAF or p300 acetyltransferase has been determined to enhance the stabilization of E2F1 by mediating its protein acetylation.^[^ ^21^, ^34^ ^]^ It is not clarified the role of KMT5C in regulating E2F1 stability whether depends on ATM activity. KMT5C may also affect the role of P/CAF or p300 in promoting E2F1 acetylation and enhancing its protein stability. This remains an interesting topic to further investigation in the future. Emerging evidence indicates that deficiencies in DNA repair response in tumor cells can active the cGAS‐STING and downstream type I IFN signaling, resulting in the transformation of immune “cold” cancers (poor immune cell infiltration) to “hot” cancers,^[^ ^26^, ^28^, ^35^ ^]^ which is in line with our findings. Similarly, we also demonstrate that either the pharmacological inhibitor A196 or the genetic inhibition of KMT5C can not only restore CD8^+^ T cell function and suppress tumor growth, but can also promote the efficacy of anti‐PD‐1 therapy in NSCLC. Previous studies have consistently reported that PARP inhibitor can activate the cGAS‐STING pathway, downstream type I IFN signaling and CCL5 secretion in NSCLC due to the induction of DNA repair deficiencies in cancer cells.^[^ ^36^, ^37^ ^]^ Although we previously find that KMT5C plays an important role in promoting liver tumor progression by activating DDR, and that KMT5C inhibition can enhance the efficacy of PARP inhibitor in liver cancer,^[^ ^16^ ^]^ its role in the regulation of tumor immunity has not been unexplored. Taken together, our current study indicates that targeting KMT5C may be a promising strategy to convert cold tumors into hot ones.

For the therapy concern, although ICB therapy has achieved encouraging clinical effects in cancer patients, only a minority of patients have received long‐term survival benefits.^[^ ^2^, ^38^ ^]^ Therefore, the optimal patient selection schemes and the development of novel combination treatment methods are still the major challenges in cancer immunotherapy field. Our current study demonstrates that KMT5C may be a potential biomarker for predicting ICB treatment response and a combination therapeutic target to enhance the efficacy of immunotherapy in NSCLC. Consistent with this role, high KMT5C expression levels in patients with NSCLC show resistance to immunotherapy and are positively correlated with a worse prognosis after ICB therapy. Similarly, either the pharmacological inhibitor A196 or the genetic inhibition of KMT5C can synergize with anti‐PD‐1 therapy in a mouse model of lung cancer. It is crucial to further assess the synergistic effect of the KMT5C inhibitors and ICB therapy in future preclinical mouse models.

Even though previously studies have showed that KMT5C plays a vital role in the progression of various human cancers, its functions are complex and depend on the cancer type.^[^ ^19^ ^]^ Currently, the clinical relevance of KMT5C in NSCLC is not investigated. In this study, we find that upregulation of KMT5C in NSCLC correlates with cancer progression and poor patient prognosis. We further demonstrate the pro‐tumor role of KMT5C in NSCLC progression in vitro and in vivo. Consistently, previously studies have reported that KMT5C promotes tumor progression in pancreatic cancer and clear cell renal cell carcinoma.^[^ ^15^, ^16^ ^]^ Similarly, we and others have revealed that KMT5C is a tumor‐promoting protein in liver cancer.^[^ ^9^, ^39^ ^]^ These findings are consistent with the current results. Besides, a recent study shows that KMT5C loss can induce EGFR inhibitor resistance in lung cancer,^[^ ^40^ ^]^ but the function of KMT5C in lung cancer progression is unexplored. In contrast, the tumor suppressor function of KMT5C is found in the breast cancer and colon cancer.^[^ ^17^, ^19^ ^]^ However, a recent study reports that KMT5C loss only promotes right‐sided colon cancer progression, but has no effect in left‐sided colon cancer.^[^ ^18^ ^]^ Thus, KMT5C exhibits tissue‐specific functions in human cancers.

In summary, our present study uncovers that KMT5C is an important promoter of NSCLC progression and immune evasion. Our findings demonstrate that the KMT5C‐mediated DNA damage response inhibits STING‐IRF3 signaling activation, resulting in the suppression of the type I IFN response, particularly CCL5 expression, leading to the downregulation of CD8^+^ T cell infiltration and function in NSCLC, which is required for the promotion of tumor immune evasion and NSCLC progression. Thus, targeting KMT5C may be a promising therapeutic approach to enhance ICB therapy in NSCLC.

## 4 Experimental Section

### Antibodies

Antibodies for western blot included: KMT5C (A16325, ABclonal, 1:1000), STING (19851‐1‐AP, Proteintech, 1:2000), p‐STING (AP1369, ABclonal, 1:800), IRF3 (A19717, ABclonal, 1:1500), p‐IRF3 (AP1412, ABclonal, 1:3000), H4K20me3 (A2372, ABclonal, 1:2000), H4 (A19815, ABclonal, 1:2000), E2F1 (A19579, ABclonal, 1:2000), ATM (#2873, Cell Signaling Technology, 1:1000), p‐ATM (#13050, Cell Signaling Technology, 1:1000), β‐Actin (A1978, Sigma‐Aldrich, 1:10 000), HRP‐conjugated Goat anti‐Rabbit IgG (AS014, ABclonal, 1:5000) and HRP‐conjugated Goat anti‐Mouse IgG (AS003, ABclonal, 1:5000).

### Cell Proliferation, Colony Formation and Cell Cycle Assays

The NSCLC cell proliferation assay was performed using the Cell Counting Kit‐8 (Dojindo, Kumamoto). A549 control cells (shNC) and KMT5C knockdown A549 cells (shKMT5C‐1 and shKMT5C‐1) were seeded in 96‐well plates with 100µl medium with 10% FBS, respectively. After 24, 48, 72 and 96 h, respectively, then cells of each well were incubated with 100ul medium with 10% CCK‐8 solution for 2 h, and then examined the absorbance at 450 nm by the multi‐label plate reader (Bio‐tek synergy HT). For colony formation assays, 300 A549 control cells and KMT5C knockdown A549 cells were respectively seeded in 12‐well plates for with 1 mL medium with 10% FBS each well for incubating 10–14 days. Then the above A549 cells were wash by PBS, and then fixed and stained, the number of the above A549 cells colonies were counted and presented as mean ± SD. The cell cycle distribution of the above A549 cells was measured using the cell cycle staining kit (MultiSciences). The data acquisition was used the fluorescence‐activated cell sorting analysis (BD Biosciences).

### Cell Migration and Invasion

The cell migration and invasion experiments of the A549 and HCC827 cells or its derivative cells were performed as the following described. The above cells were first starved for 24 h and added to the top chambers with or without matriget in a 24 transwell plate (Corning). After cell culture 24–48 h, then the above cells that migrated and invaded were respectively fixed and stained for 30 min. Excess dye from the above cells was washed off using PBS. Last, using the cotton swabs to remove the non‐migrating and non‐invading cells. The number of above cells that migrated and invaded were counted and presented as mean ± SD.

### Quantitative Real‐Time PCR and Chromatin Immunoprecipitation

The quantitative real‐time PCR and chromatin immunoprecipitation (ChIP) assays were performed as previously described.^[^ ^41^ ^]^ Using the TRIzol Reagent (Invitrogen) to extract the total RNA of A549, HCC827 and LLC cells, and the real‐time PCR experiment was performed using the Prime‐Script RT reagent Kit (TaKaRa, China) and SYBR Premix Ex Taq (TaKaRa, China), respectively according the product protocols. For chromatin immunoprecipitation (ChIP) assay was performed using the enzymatic chromatin IP kit (Cell Signaling) according to the product protocol. The sonicated chromatin fragments from A549 and LLC cells were immunoprecipitated using anti‐H4K20me3 antibody or Ig G.

### Clinical Samples and Immunohistochemistry

The clinical tissues were collected from 173 NSCLC patients in Zhongshan Hospital affiliated to Fudan University between 2014 and 2015. Seventeen pairs of NSCLC tumor tissues and non‐tumor tissues were randomly collected for the real‐time PCR assay. Eighty‐nine paraffin‐embedded NSCLC samples with anti‐PD‐1 therapy were collected from Zhongshan Hospital, including 50 therapy responder and 39 non‐responder tissues. The diagnosis of all NSCLC samples was confirmed by two independent pathologists. The informed consent from the NSCLC patients was obtained before the tissue samples collection. This research had been approved by the institutional ethical review board at Zhongshan Hospital affiliated to Fudan University (B2021‐128).

IHC staining procedures was performed as the following protocol. Primary antibodies included: Primary antibodies included: anti‐Ki67 (ab15580, Abcam, 1:1000); anti‐γH2AX (#9718, CST, 1:300); SUV420H2/KMT5C (HPA052294, Sigma, 1:300); H4K20me3 (A2372, ABclonal, 1:300); CD3 (GB111337, Servicebio, 1:200); CD8 (GB15068, Servicebio, 1:200); IFN‐γ (15365‐1‐AP, Proteintech, 1:200); GZMA (A6231, ABclonal, 1:200); GZMB (13588‐1‐AP, Proteintech, 1:200); IHC signals were detected and a relative IHC staining score (0‐8) was calculated as previously described(41).

### Cell Dissociation and Flow Cytometry Analysis

After the tumor was removed from the mice, the mouse lung cancer tissue was then washed with PBS and subsequently dissected into small pieces with scissors. Collagenase IV was used to digest the tissue on the shaker at 37 °C for ≈20 min. The digestion reaction was then terminated, and the mixture was filtered through the cell filter to remove undigested tissue fragments. To analyze immune cell infiltration in the tumor microenvironment, cell pellets from each sample were stained. Using the BD Horizon fixable viability stain 780 (FVS780) to assess cell viability. For the cell surface marker staining and intracellular staining of CD8^+^ T cells, cells were incubated with lonomycin, Phorbol 12‐Myristate 13‐Acetate and Brefeldin A for 4 h. Then, cells were stained with anti‐CD45‐PE‐Cyanine7 (BD Biosciences, Clone 30‐F11), anti‐CD3‐PE (Biolegend, Clone 145‐2C11), anti‐CD8‐PerCP/Cyanine5.5 (Biolegend, Clone 53–6.7) and anti‐CD4‐BV605 (BD Biosciences, Clone RM4‐5) antibodies for 30 min. Following washing with PBS, the cells were fixed with Fixation/Permeabilization solution (BD Biosciences) for 30 min. Then, cells were stained with anti‐IFN‐γ‐BV421 (Biolegend, Clone XMG1.2) and anti‐Granzyme B‐FITC (Biolegend, Clone QA18A28) for 30 min. Cells were collected using the LSR Fortessa flow cytometer (BD Biosciences) and the data was analyzed with FlowJo software.

### Tumor Xenograft Mouse Models

Male athymic BALB/c nude mice and male C57BL/6 mice were raised in the specific pathogen‐free conditions. The animal experiments of this study were performed at Ren Ji Hospital and also received approval by the Institutional Animal Care and Use Committee of Ren Ji Hospital, School of Medicine, Shanghai Jiao Tong University (RJ20220720).

For human lung cancer cell mouse model, A549 control cells (shNC) and KMT5C knockdown A549 cells (shKMT5C‐1 and shKMT5C‐1) were subcutaneously injected in the male athymic BALB/c nude mice (6–7 weeks old, n = 6), respectively. After 6 days post‐injection, the tumor volume of the above mice was initial measured. After about 30 days, these above mice were then anesthetized and sacrificed, and the tumor weight of these mice was examined and the tumor tissues from the above mice were used for further analysis.

For mouse lung cancer cell mouse model, the LLC control and Kmt5c knockdown cells (Control, shKmt5c‐1 and shKmt5c‐2) were subcutaneously injected in the male C57BL/6 mice (6 weeks old, n = 6), respectively. The tumor volume and weight of these above mice were examined, and the tumor tissues from these mice were used for further analysis. For tumor metastasis experiment, we used the LLC control and Kmt5c knockdown cells (Control and shKmt5c) to construct the mouse lung metastasis model in C57BL/6 mice (6 weeks old) by the tail‐vein injection. After 3–4 weeks, these mice were then anesthetized and sacrificed, the lung tissues from these above mice were collected and the metastatic nodules of these lung tissues were examined under microscope and using H&E staining.

For examining the contribution of T cells to the tumor‐suppressing effect of Kmt5c knockdown in lung cancer mouse model, the LLC control and Kmt5c knockdown cells (Control, and shKmt5c) were subcutaneously injected in the male athymic BALB/c nude mice (6 weeks old, n = 8 mice/group) and male C57BL/6 mice (6 weeks old, n = 8 mice/group), respectively. After 6 days post‐injection, the tumor volume of the above mice was initial measured. After 12 days post‐injection, these above mice were then anesthetized and sacrificed, the tumor weight of these mice were examined.

For treatment experiment, LLC cells were subcutaneously injected in the male C57BL/6 mice (6 weeks old, n = 8 mice/group, four groups), respectively. After 3–5 days post‐injection, mice were treated with vehicle control and A196 (20 mg per kg daily, intragastrically (i.g)), anti‐PD‐1 antibody (100 µg per time, every 2 days for a total of 4 intraperitoneal injection (i.p)) or a combined respectively until the end of these mouse experiments. After 5 days post‐injection, the tumor volume of the above mice was initial measured. After 13 days post‐injection, these above mice were then anesthetized and sacrificed, the tumor volume and weight of these mice were examined, and the tumor samples from these above mice were then collected for further flow cytometry and IHC analysis.

### Statistical Analysis

Statistical analysis was performed using the GraphPad Prism 9. Gene set enrichment analysis (GSEA) was performed using the GSEA v4.1.0. Kaplan‐Meier analysis and Log‐rank test were respectively used to determine survival probability and compare survival between groups. Two‐tailed unpaired Student's t‐test, two‐tailed paired Student's t‐test one‐way ANOVA and two‐way ANOVA were used for group comparisons. Data are presented as mean ± SD from at least three independent experiments, and p value <0.05 was considered statistically significant (*p < 0.05, **p < 0.01, and ***p < 0.001).

## Conflict of Interest

The authors declare no conflict of interest.

## Author Contributions

Y.Y., Q.L., G.Y., and Y.Q. contributed equally to this work. Y.Y., G.Y., Y.W., D.G., and Y.L. contributed to the design of experiments, preparation and revision of the article. Y.W., Y.Y., Q.L., G.Y., Y.Q., W.G., and S.L., performed animal studies. Y.Y., Q.L., G.Y., and Y.Q. assisted with xenograft and IHC assays. Y.Y., Q.L., G.Y., Y.Q., W.G., S.L., F.W., Z.Z., Y.W., D.G., and Y.L. contributed to the acquisition of the data. Y.Y., Q.L., G.Y., W.S., Y.W., D.G., and Y.L. contributed to the data analyze and writing of the manuscript. All authors commented on the manuscript.

## Supporting information



Supporting Information

## Data Availability

The data that support the findings of this study are available from the corresponding author upon reasonable request.
