# Supplementary material for: Targeting KMT5C Suppresses Lung Cancer Progression and Enhances the Efficacy of Immunotherapy
Source: Adv Sci (Weinh). 2025 Mar 24;12(19):2407575. doi: 10.1002/advs.202407575 (PMC12097080; doi:10.1002/advs.202407575)
Supplement: Supplementary file 1 — Supporting Information [file ADVS-12-2407575-s001.docx]

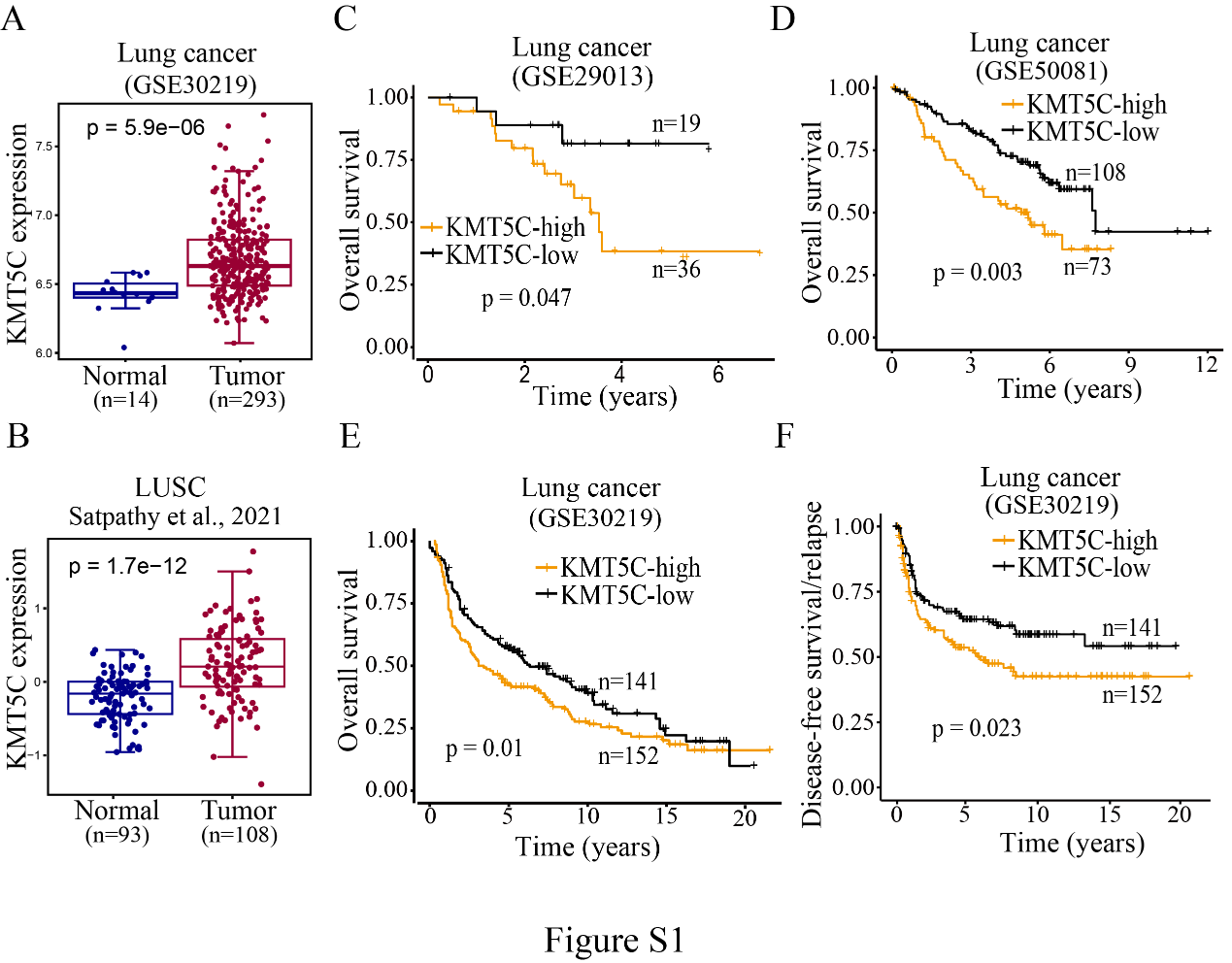


**Figure S1 The expression of KMT5C is upregulated in lung cancer and correlates with poor prognosis.**

**A**, **B** Comparison the mRNA level of KMT5C between normal and tumor tissues in the lung cancer GEO database (**A**) and LUSC cohort (**B**) as indicated. **C**, **D** Kaplan-Meier analysis of overall survival of KMT5C levels in two lung cancer cohorts (GSE29013 (**C**) and GSE50081(**D**)) as indicated. The statistical significance was assessed using log-rank test. **E**, **F** Kaplan-Meier analysis of overall survival (**E**) and disease-free survival/relapse probability (**F**) of KMT5C levels in lung cancer patients from a GEO cohort (GSE30219). The statistical significance was assessed using log-rank test. For **A** and **B**, **s**tatistical significance was calculated using two-tailed unpaired Student’s t-test.


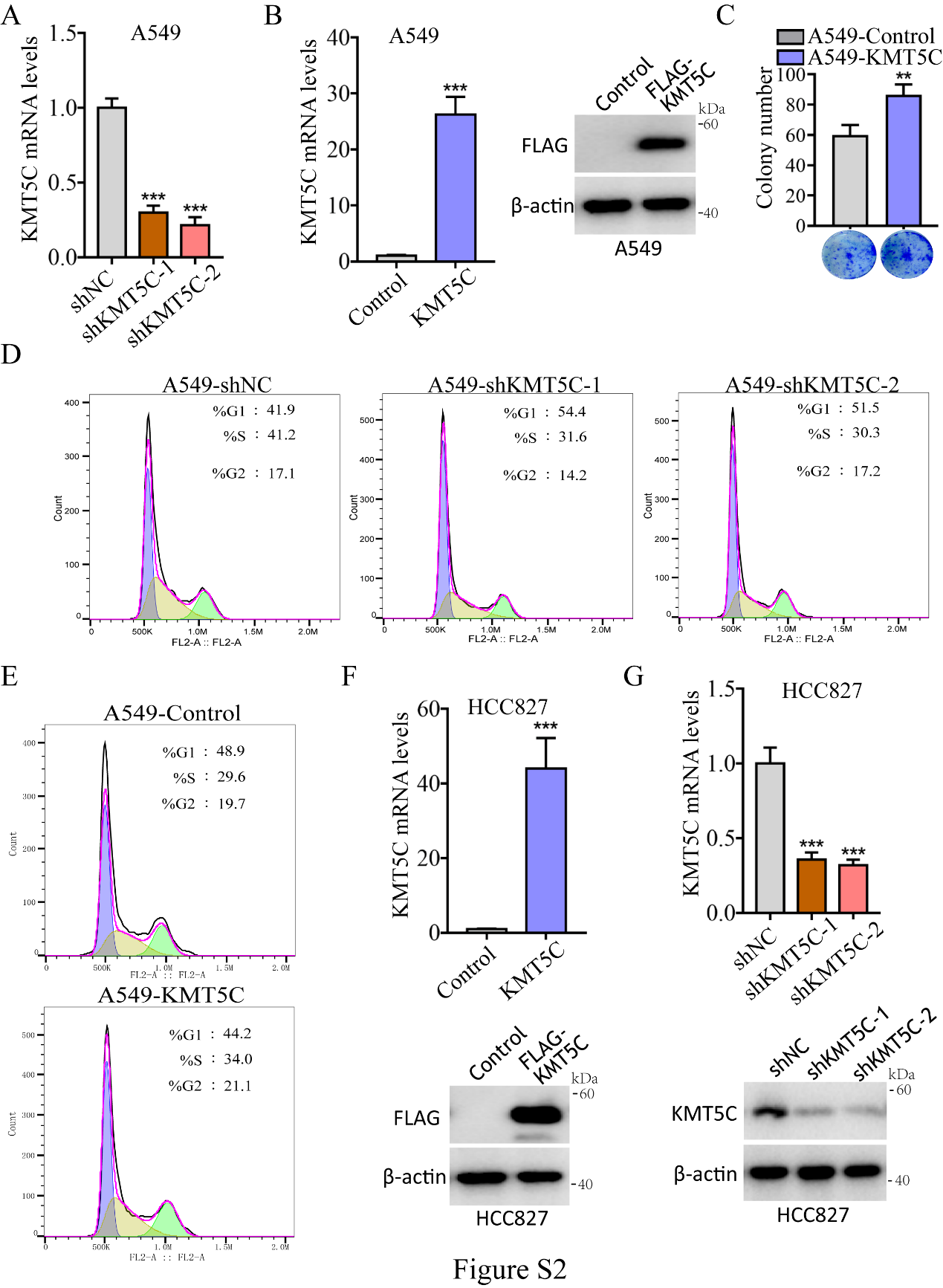


**Figure S2 KMT5C promotes NSCLC cell cycle progression.**

**A** Real-time PCR analysis the A549 cells infected with the lentivirus expressing KMT5C knockdown shRNA (shKMT5C-1 and shKMT5C-1) or control shRNA (shNC) as indicated (n=3). **B** Real-time PCR and western blot analysis the A549 cells infected with control and KMT5C overexpression lentivirus (n=3). **C** Representative images and the statistical data of colony formation from the A549 cells infected with the indicated lentivirus (n=3). **D** Representative the cell cycle images of A549 cells after stably KMT5C knockdown. **E** Representative the cell cycle images of A549 cells after KMT5C overexpression. **F** Real-time PCR and western blot analysis the HCC827 cells infected with control and KMT5C overexpression lentivirus (n=3). **G** Real-time PCR and western blot analysis the HCC827 cells infected with the lentivirus expressing KMT5C knockdown shRNA (shKMT5C-1 and shKMT5C-1) or control shRNA (shNC) as indicated (n=3). For **A** and **G**, statistical significance was calculated using one-way ANOVA. For **B**, **C** and **F**, statistical significance was calculated using two-tailed unpaired Student’s t-test. Data are presented as mean ± SD and **P<0.01, ***P<0.001.


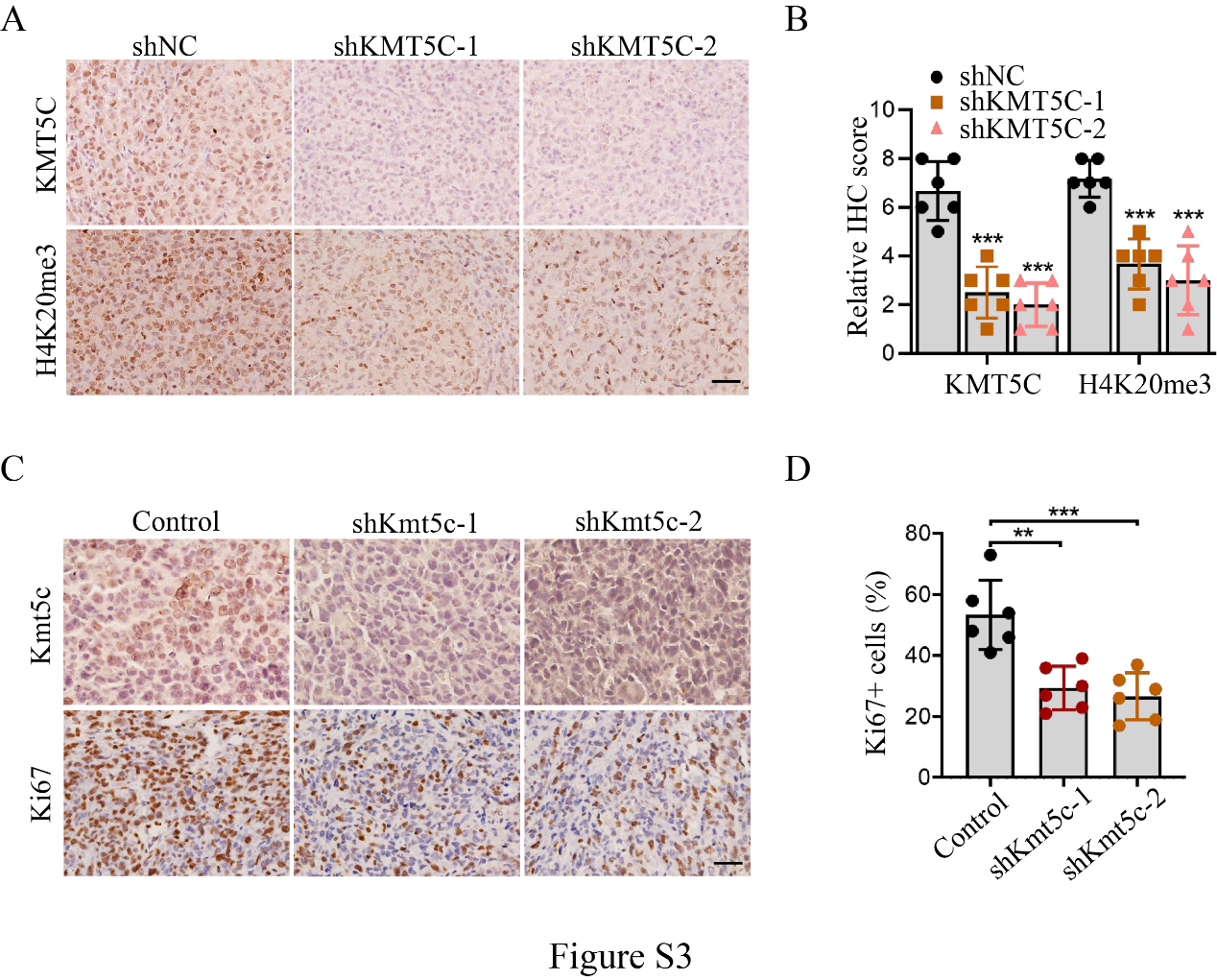


**Figure S3 Knockdown of KMT5C affects NSCLC tumor growth.**

**A**, **B** Representative images of IHC KMT5C and H4K20me3 staining (**A**), and the relative IHC scores of KMT5C and H4K20me3 expression (**B**) in the tumor tissues as indicated (n=6). Scale bar, 50 µm. **C**, **D** Representative images of IHC Kmt5c and Ki67 staining (**C**), and the relative IHC scores of Ki67 expression (**D**) in the tumor tissues as indicated (n=6). Scale bar, 50 µm. Statistical significance was calculated using one-way ANOVA. Data are presented as mean ± SD and **P<0.01, ***P<0.001.


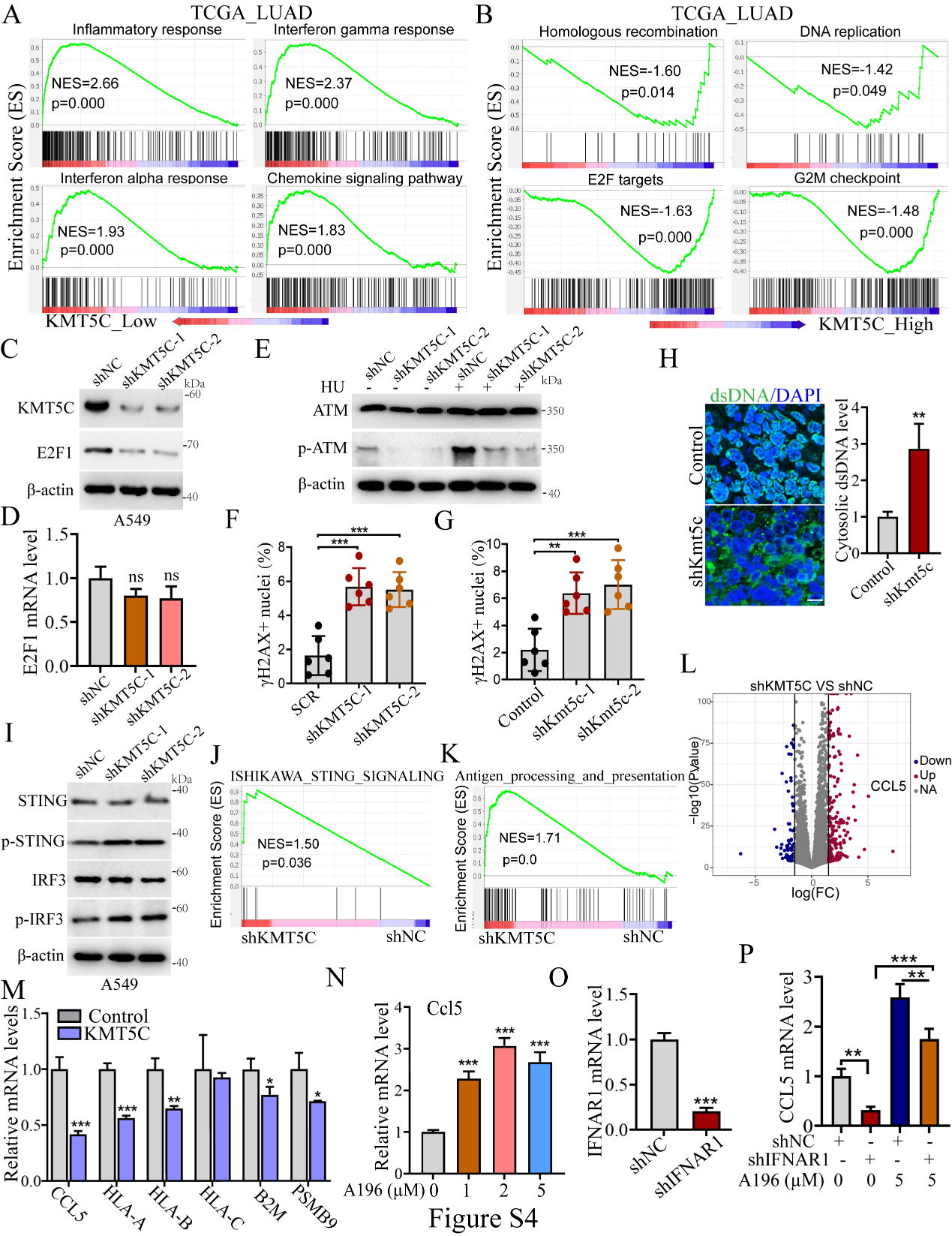


**Figure S4 KMT5C knockdown activates the STING-IRF3 signaling and promotes CCL5 expression in NSCLC.**

**A**, **B** GSEA shows the interferon-related signaling enriched in KMT5C low expression group (**A**) and DDR pathways enriched in KMT5C high expression group (**B**). Statistical significance was calculated using Permutation test. **C** Western blot analysis the expression of E2F1 in the A549 cells infected with lentivirus as indicated. **D** Real-time PCR analysis the expression of E2F1 in A549 cells infected with the lentivirus as indicated (n=3). **E** Western blot analysis the expression levels of ATM and p-ATM in the A549 cells after hydroxyurea (HU, 2mM) treatment or without as indicated. **F** The IHC scores of γH2AX expression in tumor tissues from A549-shNC or A549-shKMT5C-1&-2 mice group (n=6). **G** The IHC scores of γH2AX expression in tumor tissues from LLC-shKmt5c or Control mice group (n=6). **H** Confocal images and quantification of cytosolic dsDNA in tumor tissues from LLC-shKmt5c or Control mice group (n=3). **I** Western blot analysis the expression levels of STING, p-STING, IRF3 and p-IRF3 in the A549 cells infected with lentivirus as indicated. **J**, **K** GSEA shows the ISHIKAWA_STING_SIGNALING (**J**) and antigen_processing_and_presentation (**K**) gene sets enriched in KMT5C knockdown group. Statistical significance was calculated using Permutation test. **L** The volcano plot shows the CCL5 top enriched in the differentially expressed genes in RNA-seq data from A549 cells upon KMT5C knockdown. **M** Real-time PCR analysis the relative mRNA levels of CCL5, HLA-A, HLA-B, HLA-C, B2M and PSMB9 from HCC827 cells after stably KMT5C overexpression as indicated (n=3). **N** Real-time PCR analysis the relative mRNA levels of CCL5 in LLC cells with or without A196 treatment as indicated (n=3). **O** Real-time PCR analysis the expression of IFNAR1 in A549 cells infected with the lentivirus as indicated (n=3). **P** Real-time PCR analysis the relative mRNA levels of CCL5 in A549 cells with or without A196 treatment as indicated (n=3). For **D**, **F**, **G**, **N** and **P**, statistical significance was calculated using one-way ANOVA. For **H**, **M** and **O**, statistical significance was calculated using two-tailed unpaired Student’s t-test. Data are presented as mean ± SD and ns. not significant, *P<0.05, **P<0.01, ***P<0.001.


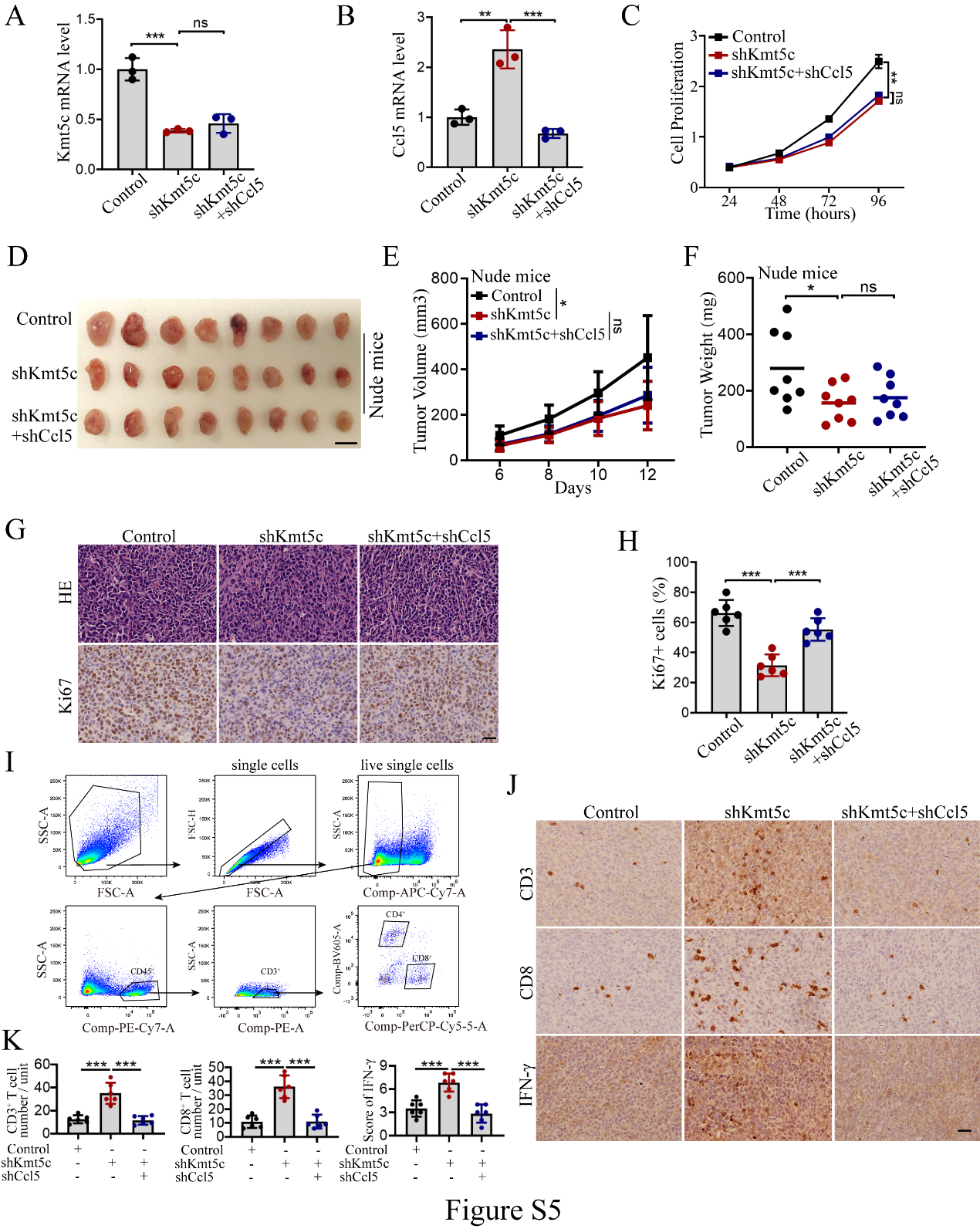


**Figure S5 Kmt5c knockdown in NSCLC promotes the infiltration and cytotoxic function of CD8^+^ T cells mainly depending on Ccl5.**

**A**, **B** Real-time PCR analysis the expression of Kmt5c (**A**) and Ccl5 (**B**) in LLC cells infected with the lentivirus as indicated (n=3). **C** CCK8 assays analysis the cell proliferation from LLC cells infected with the indicated lentivirus (n=4). **D-F** LLC cells stably expressing the indicated shRNA were subcutaneously injected in nude mice respectively. Shown are the representative image of tumors (**D**), and statistical data of tumor volume (**E**) and weight (**F**) as the indicated (n=8). Scale bar, 1 cm. **G**, **H** Representative images of HE and IHC Ki67 staining (**G**), and the IHC scores of Ki67 expression (**H**) in the tumor tissues as indicated (n=6). Scale bar, 50 µm. **I** Schematic representation of flow cytometric approach and sequential gates. **J**, **K** Representative images of IHC CD3, CD8 and IFN-γ staining (**J**) and the statistical data (**K**) of numbers of the positive cells of CD3 and CD8, and the IHC scores of IFN-γ expression in the tumor tissues of mouse as indicated (n=6). Scale bar, 50 μm. Data are presented as mean ± SD. For **C** and **E**, statistical significance was calculated using two-way ANOVA. For **A**, **B**, **F**, **H** and **K**, statistical significance was calculated using one-way ANOVA. ns. not significant, *P<0.05, **P<0.01, ***P<0.001.


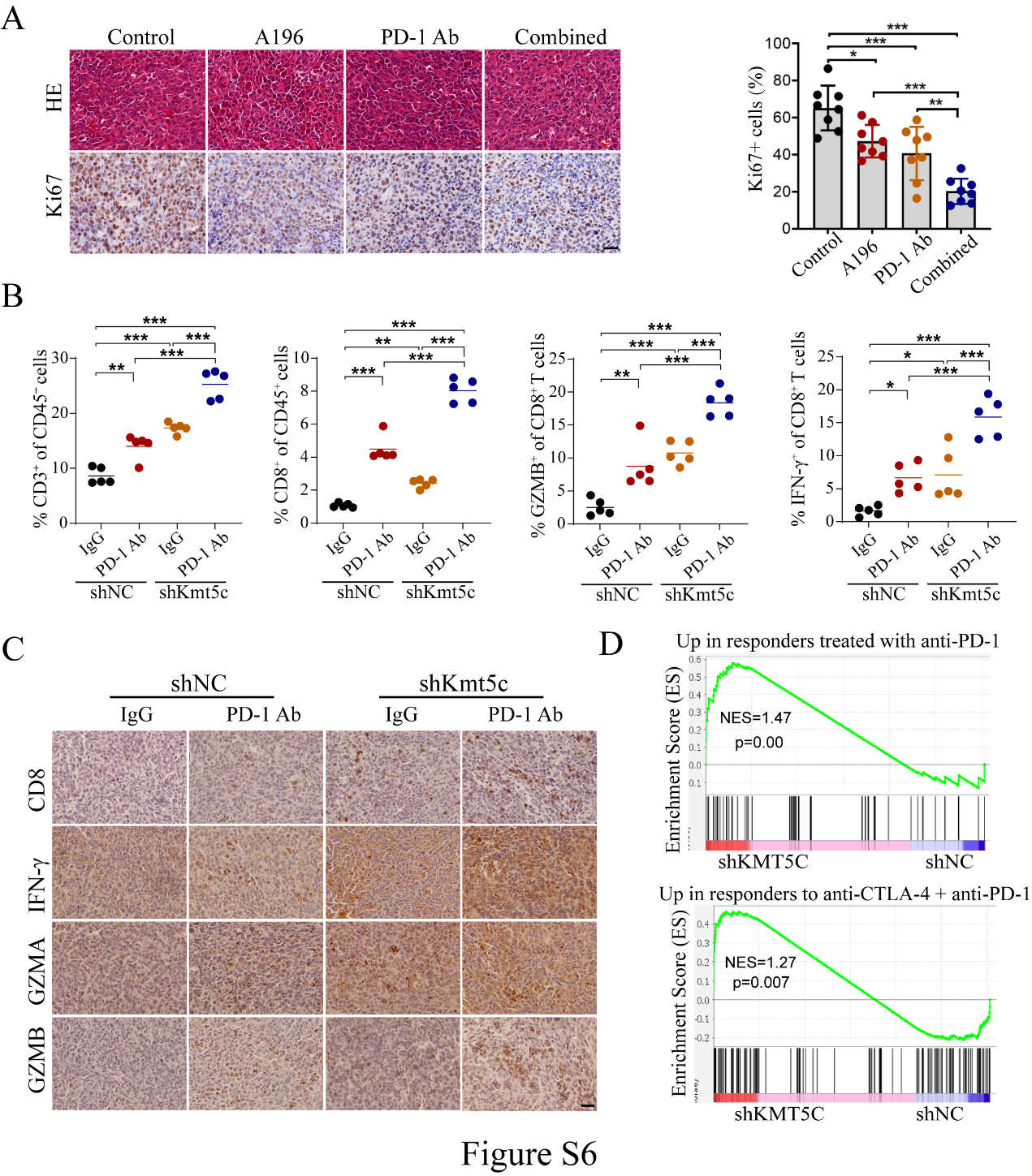


**Figure S6 Kmt5c knockdown enhances the response of NSCLC to anti-PD-1 therapy.**

**A** Representative image of HE and IHC Ki67 staining, and the IHC scores of Ki67 expression in the tumor tissues as indicated (n=8). Scale bar, 50 µm. **B** Relative proportions of CD3^+^ and CD8^+^ T cells, and IFN-γ^+^ and GAMB^+^ of CD8^+^ T cells in tumor tissues were analyzed by flow cytometry (n=5). **C** Representative images of IHC CD8, IFN-γ, GZMA and GAMB staining in the tumor tissues as indicated. Scale bar, 50 µm. **D** GSEA shows the gene sets of up in response to anti-PD-1 or the combination of anti-CTLA-4 and anti-PD-1 therapies enriched in KMT5C knockdown group as indicated. Statistical significance was calculated using Permutation test. For **A** and **B**, statistical significance was calculated using one-way ANOVA. Data are presented as mean ± SD and *P<0.05, **P<0.01, ***P<0.001.


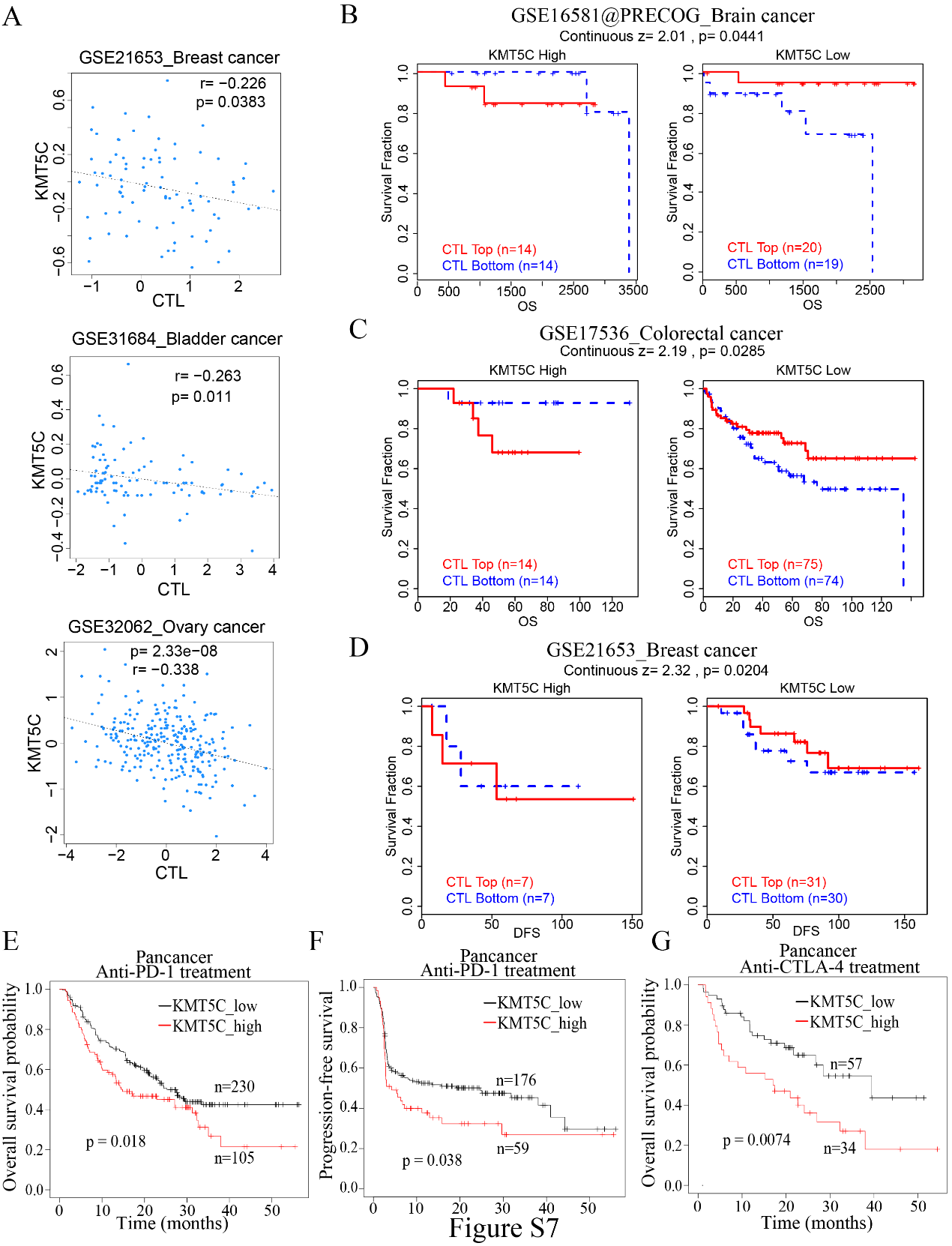


**Figure S7 High expression of KMT5C correlates with worse prognosis to ICB therapy in human cancers.**

**A** Correlation analysis of the cytotoxic T lymphocyte (CTL) level and the expression of KMT5C in breast cancer (GSE21653), bladder cancer (GSE31684) and ovary cancer (GSE32062) cohorts from the TIDE database (<http://tide.dfci.harvard.edu/>). The Pearson coefficient was used to evaluate correlations. **B**-**D** Kaplan-Meier analysis of the association between CTL levels with overall survival (OS) in brain cancer (**B**) and colorectal cancer (**C**) cohorts, and with the disease-free survival (DFS) in breast cancer patients (**D**) with KMT5C high or low expression group from the TIDE database (<http://tide.dfci.harvard.edu/>). **E**, **F** Survival analysis of overall survival probability (**E**) and progression-free survival (**F**) of KMT5C levels in pancancer patients receiving anti-PD-1 therapy from KM plotter (https://kmplot.com/analysis/index.php?p=background).

The statistical significance was assessed using log-rank test. **G** Survival analysis of overall survival probability of KMT5C levels in pancancer patients receiving anti-CTLA-4 treatment from KM plotter. The statistical significance was assessed using log-rank test.
